# Bezafibrate Upregulates Mitochondrial Biogenesis and Influence Neural Differentiation of Human-Induced Pluripotent Stem Cells

**DOI:** 10.1007/s12035-018-1368-2

**Published:** 2018-10-13

**Authors:** Justyna Augustyniak, Jacek Lenart, Paweł Gaj, Monika Kolanowska, Krystian Jazdzewski, Piotr Pawel Stepien, Leonora Buzanska

**Affiliations:** 1 Stem Cell Bioengineering Unit, Mossakowski Medical Research Centre Polish Academy of Sciences, Warsaw, Poland; 2 Department of Neurochemistry, Mossakowski Medical Research Centre Polish Academy of Sciences, Warsaw, Poland; 3 Laboratory of Human Cancer Genetics, Centre of New Technologies, University of Warsaw, Warsaw, Poland; 4 Genomic Medicine, Medical University of Warsaw, Warsaw, Poland; 5 Institute of Genetics and Biotechnology, Faculty of Biology, University of Warsaw, Warsaw, Poland; 6 Institute of Biochemistry and Biophysics, Polish Academy of Sciences, Warsaw, Poland; 7 Centre of New Technologies, University of Warsaw, Warsaw, Poland

**Keywords:** Bezafibrate, Mitochondrial biogenesis, hiPSC, NSC, PPAR’s, PGC-1α

## Abstract

Bezafibrate (BZ) regulates mitochondrial biogenesis by activation of PPAR’s receptors and enhancing the level of PGC-1α coactivator. In this report, we investigated the effect of BZ on the expression of genes (1) that are linked to different pathways involved in mitochondrial biogenesis, e.g., regulated by PPAR’s receptors or PGC-1α coactivator, and (2) involved in neuronal or astroglial fate, during neural differentiation of hiPSC. The tested cell populations included hiPSC-derived neural stem cells (NSC), early neural progenitors (eNP), and neural progenitors (NP). RNA-seq analysis showed the expression of *PPARA*, *PPARD* receptors and excluded *PPARG* in all tested populations*.* The expression of *PPARGC1A* encoding PGC-1α was dependent on the stage of differentiation: NSC, eNP, and NP differed significantly as compared to hiPSC. In addition, BZ-evoked upregulation of *PPARGC1A*, *GFAP*, *S100B*, and *DCX* genes coexist with downregulation of *MAP2* gene only at the eNP stage of differentiation. In the second task, we investigated the cell sensitivity and mitochondrial biogenesis upon BZ treatment. BZ influenced the cell viability, ROS level, mitochondrial membrane potential, and total cell number in concentration- and stage of differentiation-dependent manner. Induction of mitochondrial biogenesis evoked by BZ determined by the changes in the level of SDHA and COX-1 protein, and mtDNA copy number, as well as the expression of *NRF1*, *PPARGC1A*, and *TFAM* genes, was detected only at NP stage for all tested markers. Thus, developmental stage-specific sensitivity to BZ of neurally differentiating hiPSC can be linked to mitochondrial biogenesis, while fate commitment decisions to PGC-1α (encoded by *PPARGC1A*) pathway.

**Electronic supplementary material:**

The online version of this article (10.1007/s12035-018-1368-2) contains supplementary material, which is available to authorized users.

## Introduction

The fibrate groups of drugs, derivatives of dehydrocholic acid, are clinically used in the treatment of lipid disorders [1, 2]. Clofibrate, gemfibrozil, bezafibrate, fenofibrate, and ciprofibrate are therapeutically useful examples of drugs belonging to fibrate family [2]. The effect of fibrates on fatty acid and lipoprotein metabolism was examined in detail in the liver, muscle, both skeletal and cardiac, and kidney [2]. Bezafibrate belongs to the fibrates which are a class of amphipathic carboxylic acids, which have agonist activity for peroxisome proliferator-activated receptor (PPAR)α, nuclear receptors involved in the transcription of genes involved in fatty acid oxidation, apolipoprotein production, and cholesterol transport, with additional functions in inflammation, endothelial function, and vascular remodeling [3].

Bezafibrate (BZ) was used in this study due to its identified influence on mitochondrial biogenesis [4]; however, information on the effect of BZ on the process of neural cell differentiation is very scarce, only a few publications describing the influence of this group of compounds on the nervous system [5].

The aim of our work was to check whether BZ, through the stimulation of mitochondrial biogenesis, can modulate the process of differentiation of neural cells at early stages of neural differentiation. At the molecular level, the effects of BZ are mediated by the activation of PPAR receptors that regulate the expression of genes involved not only in lipid homeostasis and energy metabolism but also many other processes, including inflammation, cellular differentiation, and proliferation [2]. PAR-peroxisome proliferator-activated receptor gamma co-activator 1-alpha (PGC-1α) encoded by *PPARGC1A* gene is considered the major regulator of mitochondrial biogenesis, also playing a role in the regulation of expression of antioxidant defenses [6–8]. Considering that PGC-1α leads to mitochondrial biogenesis, several studies have evaluated BZ as a potential pharmacological strategy for neurodegenerative disorders characterized by mitochondrial dysfunction. Human-induced pluripotent stem cells (hiPSC) hold great potential in the field of regenerative medicine, disease modeling, and drug screening. More and more evidence shows that mitochondria play a fundamental role in the process of differentiation. hiPSC rely mainly on aerobic glycolysis for energy production, and mitochondria display an immature phenotype and reduced activity. Upon the initiation of differentiation, a switch from glycolysis to oxidative phosphorylation occurs in the differentiating cells because the more specialized cells have a greater demand for ATP. mtDNA copy number seems to be an important factor for the appropriate initiation of differentiation.

**Fig. 1:**
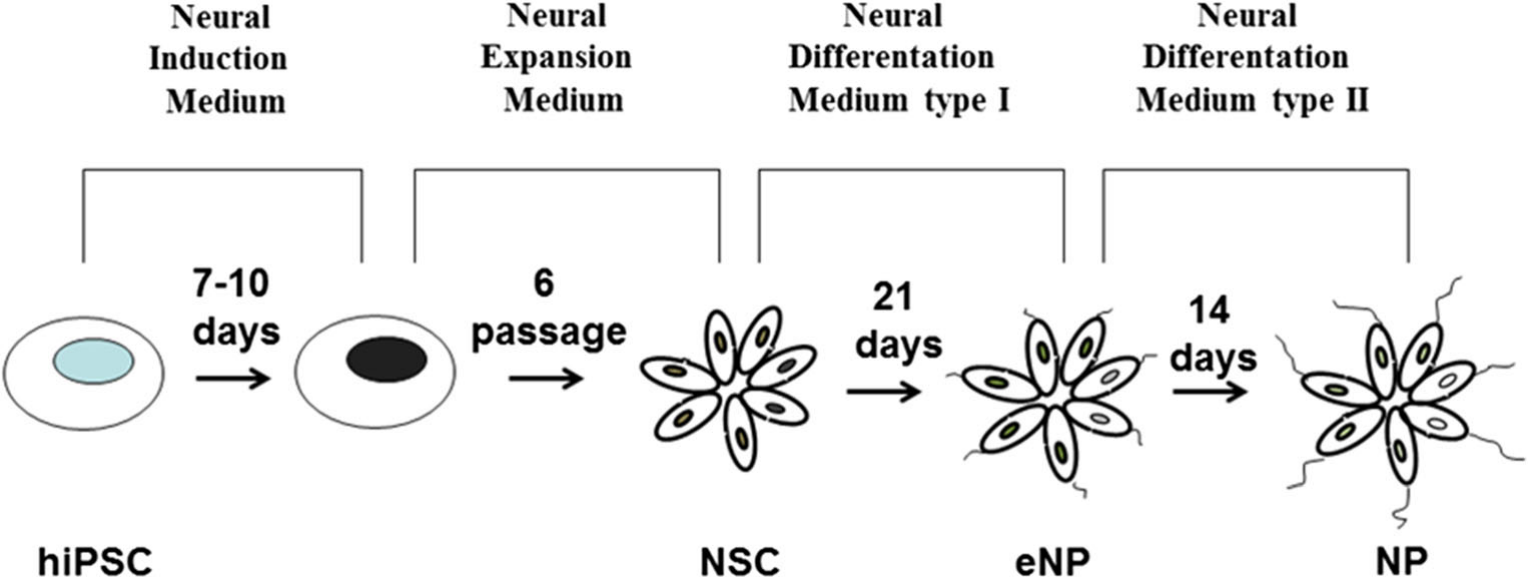
Protocol for differentiation of hiPSC into three stages of the early neural development: neural stem cells (NSC), early neural progenitors (eNP), and neural progenitors (NP)

The starting population of hiPSC present the phenotype of ESC-like state with high self-renewal and differentiation potency in vitro and in vivo*.* In the defined culture condition, hiPSC have the ability to differentiate into neurons, astrocytes, and oligodendrocytes [9, 10]. In our in vitro study, we used neural stem cells (NSC), early neural progenitors (eNP), and neural progenitors (NP) derived from hiPSC (Fig. 1). We have shown that three cell populations obtained during early neural differentiation of hiPSC reveal distinct characteristic and differ significantly on the level of transcription of genes encoding pluripotency and neural differentiation markers. The cell phenotype was confirmed by immunofluorescence staining, RT-PCR, and RNA-seq [11, 12].

In this report, we aimed to answer the question whether upregulation of mitochondrial biogenesis by BZ in hiPSC can be related to the regulation of their neural fate commitment. Based on RNA-seq data, we investigated the expression of genes that are linked to different pathways involved in mitochondrial biogenesis, e.g., regulated by PPAR’s receptors or PGC-1α coactivator, during neural differentiation of hiPSC. We tested also the influence of three different concentrations of BZ on the viability, mitochondrial membrane potential, ROS level, total cell number, and mitochondrial biogenesis revealed by the level of SDHA and COX-1 protein. The efficient highest concentration of BZ was further chosen to test mitochondrial biogenesis at mRNA level (*NRF1, TFAM, PPARGC1A*) gene expression (qRT-PCR) and mtDNA copy number (qPCR) assay. The phenotypic changes during neural differentiation were controlled by the analysis of gene expression (qRT-PCR) of the main neuronal markers: *MAP2, DCX*, and the astrocytic markers: *GFAP, S100B*. Our results indicated that upregulation of mitochondrial biogenesis and sensitivity to BZ of hiPSC is dependent upon the stage of neural development, while fate commitment decisions are linked to PGC-1α (encoded by PPARGC1A) pathway.

## Materials and Methods

### Cell Culture and BZ Treatment

hiPSC (Gibco® Human Episomal iPSC Line, Life Technologies, Thermo Fisher Scientific) were differentiated into NSC, eNP, and late NP in accordance with the protocol of [13]) with some modifications as described previously [11, 12]. hiPSC at the undifferentiated stage of development were cultured in Essential 8 Medium (Thermo Fisher Scientific) on a 6-well plate covered with rh-Vitronectin (Thermo Fisher Scientific) with medium replaced every day. For the experiments, the cells were seeded on a plate covered with a solution of Matrigel: DMEM/F12, 1:30 ratio (BD Matrigel™ Basement Membrane Matrix, BD Biosciences) at density 5 × 105 cells/cm^2^ on 6-well, 24-well, or 96-well (Nunc) plates. On the next day, cells were treated with BZ (Sigma-Aldrich) in the concentrations 0 μM, 12.5 μM, 25 μM, and 50 μM in medium dedicated to the neural stage of differentiation: NSC (Neurobasal, Neural Induction Supplement 1:50, Advanced DMEM, 1:1, AAS 1:100), eNP (Neurobasal, DMEM/F12 [1:1], N2 supplement 1%, B27 supplement 1%, EGF [20 ng/mL], bFGF [20 ng/mL]), and NP (Neurobasal, DMEM/F12 [1:1], N2 supplement 1%, B27 supplement 1%). After 5 days of exposition to BZ, samples were taken to evaluate its effects on the cells at the specific stage of development. The phenotype of the control populations used in the experiments of this study was characterized before exposition to BZ by RT-PCR, RNA-seq, and immunocytochemistry staining methods as previously described [11, 12]

### Alamar Blue Viability Assay

Cell viability was measured on the fifth day of BZ exposition at the following concentration range: 0 μM, 12.5 μM, 25 μM, 50 μM. Afterwards, the medium was changed to fresh one containing resorufin (1:10 ratio). After 3 h of incubation, the fluorescence at excitation 544 nm and emission 590 nm wave lengths were measured using a plate reader (Fluoroscan Ascent, FL, Labsystems). Data were normalized to total cell number with Janus green staining according to the manufacturer’s protocol. The results are shown as the [%] of control.

### ROS Level

After 5 days of exposure to BZ, the medium was changed to fresh one containing (1 μM) of DCFHDA (dichloro-dihydro-fluorescein diacetate, Sigma-Aldrich). Three hours later, the fluorescence intensity (DCF) (485 nm excitation–538 nm emission) was recorded using a plate reader (Fluoroscan Ascent FL, Labsystems) as described previously [11, 12]. Normalization of data to the total cell number was performed by Janus green staining according to the manufacturer’s protocol. Results were calculated as the ratio [%] of the test samples to the untreated control.

### Mitochondrial Membrane Potential

Mitotracker® Red CMXRos (Thermo Fisher Scientific) staining was used to determine mitochondrial membrane potential in NSC, eNP, and NP populations exposed to BZ as described previously [11, 12]. On day 5, the medium was changed to fresh one supplemented with 50 nM Mitotracker® Red CMXRos. Four hours later, fluorescence at excitation 579 nm and emission 599 nm was measured with a plate reader (Fluoroscan Ascent FL, Labsystems). The results are shown as the ratio [%] of the test sample to the untreated control after normalization to cell number with Janus green staining according to the manufacturer’s protocol.

### SDHA, COX-1 Protein Level, and Proliferation Ratio Determination

Levels of SDHA and COX-1 (MT-CO1) proteins were evaluated with MitoBiogenesis™ In-Cell ELISA Kit (Abcam) on Fluostar plate reader (OMEGA, BMG LABTECH), according to the manufacture’s protocol, after 5 days of exposure to BZ. The results are shown in the graph as the [%] of the untreated control. The total number of cells was determined with Janus green and calculated from a standard curve. The details of the measurement have been described previously [11, 12].

### Isolation of Nucleic Acids

Total RNA and DNA were extracted from NSC, eNP, and NP cells using ZR-DuetTM DNA/RNA MiniPrep Kit (Zymo Rsearch). RNA was purified using Clean-Up RNA Concentrator kit (A&A Biotechnology). After purification, RNA integrity was assessed by electrophoresis on 2% agarose gels. The concentration of RNA and DNA was determined by using NanoDrop ND-1000 spectrophotometer (Thermo Fisher Scientific). The DNA was used in the mtDNA copy number analysis (qPCR). The RNA was converted into cDNA in the RT reaction (High-Capacity RNA-to-cDNA™ Kit (Thermo Fisher Scientific) on Civic Cycler Thermal cycler (Biotech INC). cDNA then was used to analyze gene expression (qRT-PCR).

### Primer Design

All primers used in these studies base on the human reference genome GRCh38 and were designed, with Primer-BLAST software (https://www.ncbi.nlm.nih.gov/tools/primer-blast/), and analyzed in silico and in vitro. Sequences of primers used here were described previously [11, 12]. Experimentally determined PCR efficiency E (%) was in the range 1.92–2.15.

### qPCR

For qPCR, 10 ng of DNA was loaded with 0.25 μM of forward and reverse primers 12.5 μL of iTaq™.

Universal SYBR® Green Supermix (Bio-rad) onto a 96-well plate for LightCycler® 96 (Roche Diagnostics GmbH) at the PCR reaction conditions: hot start at 95 °C for 3 min followed by 45 cycles of denaturation at 95 °C for 10 s, annealing at 60 °C for 30 s, and extension at 72 °C for 30 s. The mtDNA copy number was calculated as (1) *ND1/SCLO2B1* and (2) *ND5/SERPINA1* ratio on the quantification cycle (Cq) values and the baseline settings automatically calculated by the qPCR instrument software. Sequences of primers used here are shown in Table 1.

**Table 1:** Primers used for qPCR

Gene symbol	GenBank number	Primer sequence	Amplicon length (bp)
*ND1 F*	NC_012920.1	TACGGGCTACTACAACCCTTC	77
*ND1 R*		ATGGTAGATGTGGCGGGTTT	
*ND5 F*	NC_011137.1	CATTACTAACAACATTTCCCCCGC	70
*ND5 R*		GGCTGTGAGTTTTAGGTAGAGGG	
*SERPINA1 F*	NM_000295.4	CAGTGAATAAATGAGGCGTACATCC	89
*SERPINA1 R*		GACTGTTTCTCATGCCTCTGGAAAG	
*SLCO2B1 F*	NM_007256.4	CCTGATGCCTAGGTTTCTTTTCTTG	85
*SLCO2B1 R*		GGTCATCTGCCTACCCTAGAAC	

*F* forward, *R* reverse

### qRT-PCR

For qRT-PCR, 10 ng of cDNA was loaded with 0.25 μM of forward and reverse primers; 12.5 μL of iTaq™ Universal SYBR® Green Supermix (Bio-rad) onto a 96-well plate for LightCycler® 96 (Roche Diagnostics GmbH) in the following steps: initial denaturation step at 95 °C for 3 min, 45 cycles of denaturation at 95 °C for 10s, and annealing/extension at 58 °C for 1 min. Samples were tested in four replicates. The Cq values automatically calculated by the qPCR instrument software were then used for data analysis GeneEx 6.1 software (MultiD Analyses AB). Relative gene expression was determined using the ΔΔCT method [14]. NormFinder was used for reference gene prediction (Fig. 6). Sequences of primers used in this experiments are shown in Table 2.

**Table 2:** Primers used for RT-qPCR

Primers	GenBank number	Primer sequence	Amplicon length
*ACTB F*	NM_001101.3	GCTCACCATGGATGATGATATCGC	169
*ACTB R*		CACATAGGAATCCTTCTGACCCAT	
*CAPN10 F*	NM_023083.3	TCTCACCGGGCTACTACCTG	86
*CAPN10 R*		CCCGGTAGAGAAGACTCGGA	
*CCNG1 F*	NM_004060.3	GCCTCTCGGATCTGATATCGT	138
*CCNG1 R*		CATTCAGCTGGTGTAGCAGT	
*DCX F*	NM_000555.3	GGAAAGGGTTTGATGAATAGCACAA	122
*DCX R*		AAGCCCTCTTTCCTACTCTAATGTG	
*EEF1A1 F*	NM_001402.5	TGTTCCTTTGGTCAACACCGA	122
*EEF1A1 R*		ACAACCCTATTCTCCACCCA	
*EID2 F*	NM_153232.3	GGCATCGCTCTGTCCAGTTA	74
*EID2 R*		GCTTGGACATCTCAGACCGT	
*GAPDH F*	NM_002046.5	GTTCGACAGTCAGCCGCATC	90
*GAPDH R*		TCCGTTGACTCCGACCTTCA	
*GFAP F*	NM_002055.4	GTGAAGACCGTGGAGATGCG	76
*GFAP R*		TGCCTCACATCACATCCTTGT	
*HPRT1 F*	NM_000194.2	AGGCGAACCTCTCGGCTTTC	166
*HPRT1 R*		CTGGTTCATCATCACTAATCACGAC	
*MAP2 F*	NM_002374.3	TGCCTCAGAACAGACTGTCAC	101
*MAP2 R*		AAGGCTCAGCTGTAGAGGGA	
*MYC F*	NM_002467.4	CCCTCCACTCGGAAGGACTA	96
*MYC R*		GCTGGTGCATTTTCGGTTGT	
*NAT1 F*	NM_001160170.3	TGGTTGCCGGCTGAAATAAC	93
*NAT1 R*		TCTGTCTAGGCCAGTCTCCT	
*NRF1 F*	NM_001001928.2	CAGCCGCTCTGAGAACTTCAT	148
*NRF1 R*		GTCTTCATCAGCACTCAGCATACTA	
*PHB F*	NM_001281496.1	TGGAAGCAGGTGAGAATGGAG	76
*PHB R*		ATCATGGAGCAGAGGAGGACT	
*PPARGC1A F*	NM_003201.2	TAGTAAGACAGGTGCCTTCAGTTC	174
*PPARGC1A R*		CTCGATGTCACTCCATACAGACTC	
*RABEP2 F*	NM_024816.2	AGGAAGGGGCAAATGGTGAG	96
*RABEP2 R*		CAGCCTTCATGGTTTCCATTTCTG	
*RPLP0 F*	NM_001002.3	CCTCGTGGAAGTGACATCGT	76
*RPLP0 R*		CTGTCTTCCCTGGGCATCAC	
*S100B F*	NM_006272.2	CGGAGGGAACCCTGACTACA	132
*S100B R*		TCTGCATGGATGAGGAACGC	
*TBP F*	NM_003194.4	GCAAGGGTTTCTGGTTTGCC	80
*TBP R*		CAAGCCCTGAGCGTAAGGTG	
*TFAM F*	NM_005011.4	TGAAAGATTCCAAGAAGCTAAGGGT	132
*TFAM R*		TAACGAGTTTCGTCCTCTTTAGCAT	
*TUBB3 F*	NM_006086.3	CAACCAGATCGGGGCCAAGTT	146
*TUBB3 R*		GAGGCACGTACTTGTGAGAAGA	
*UBC F*	NM_021009.6	ACGGGACTTGGGTGACTCTA	82
*UBC R*		ATCGCCGAGAAGGGACTACT	
*ZNF324B F*	NM_207395.2	CATTGGAAGGACAAACCTAGGATGATG	164
*ZNF324B R*		CTTATCTGCTCCAAAGCTATCACTGTC	

*F* forward, *R* reverse

### RNA-seq

Sequencing was performed with the use of NextSeq 500/550 High Output Kit-75 cycles (cat. no. FC-404-2005, Illumina, USA) on NextSeq 500 sequencer (Illumina, USA). The libraries were subjected to sequencing using the Illumina NextSeq 500 76 bp single-end mode. Before using in statistical analysis, the raw RNA-seq data (FASTQ) was trimmed for adapter sequences and filtered using Trimmomatic [15]. Then the reads were mapped with STAR [16] (aligner using the reference genome (GRCh38) coupled with Ensembl 88 transcriptome annotations). Transcription levels were calculated using HTseq-count [17, 18] and were further used for differential gene expression (DGE) analysis using edgeR [19]. Data are presented as normalized counts per million (log2) and differential gene expression results.

The details of the measurement have been described previously [11, 12]. The expression of genes participating in mitochondrial biogenesis regulation and the PPAR and PGC-1α signaling pathways is presented in the form of “heat map” which was built based on the software MORPHEUS (Broad Institute, https://software.broadinstitute.org/morpheus/)*.*

### Analysis In Silico

In silico analysis with Genemania web-tool (https://genemania.org/*)* was applied to build up predicted network of interactions between genes involved in PGC-1α pathway and neuronal differentiation. The performed analysis and detailed sources are provided in Supplementary Data 2.

## Statistical Analysis

Statistical analysis of the results was carried out using the GraphPad Prism 5.0. (LaJola, USA) software. The Kolmogorov-Smirnov test was used as the normality assay. The statistical significance between samples vs. control was obtained with Student’s *t* test or one-way ANOVA with Tukey’s post-test. A statistical significance between groups was obtained with two-way ANOVA, with Bonferroni post-test (*), *p* < 0. 05. Results represent three independent experiments, each at least in four replicates. Results shown in the brackets were obtained as mean with (± SEM). Data in text were presented as mean with standard deviation (± SD).

## Results

### Characterization of Cell Populations

**Fig. 2:**
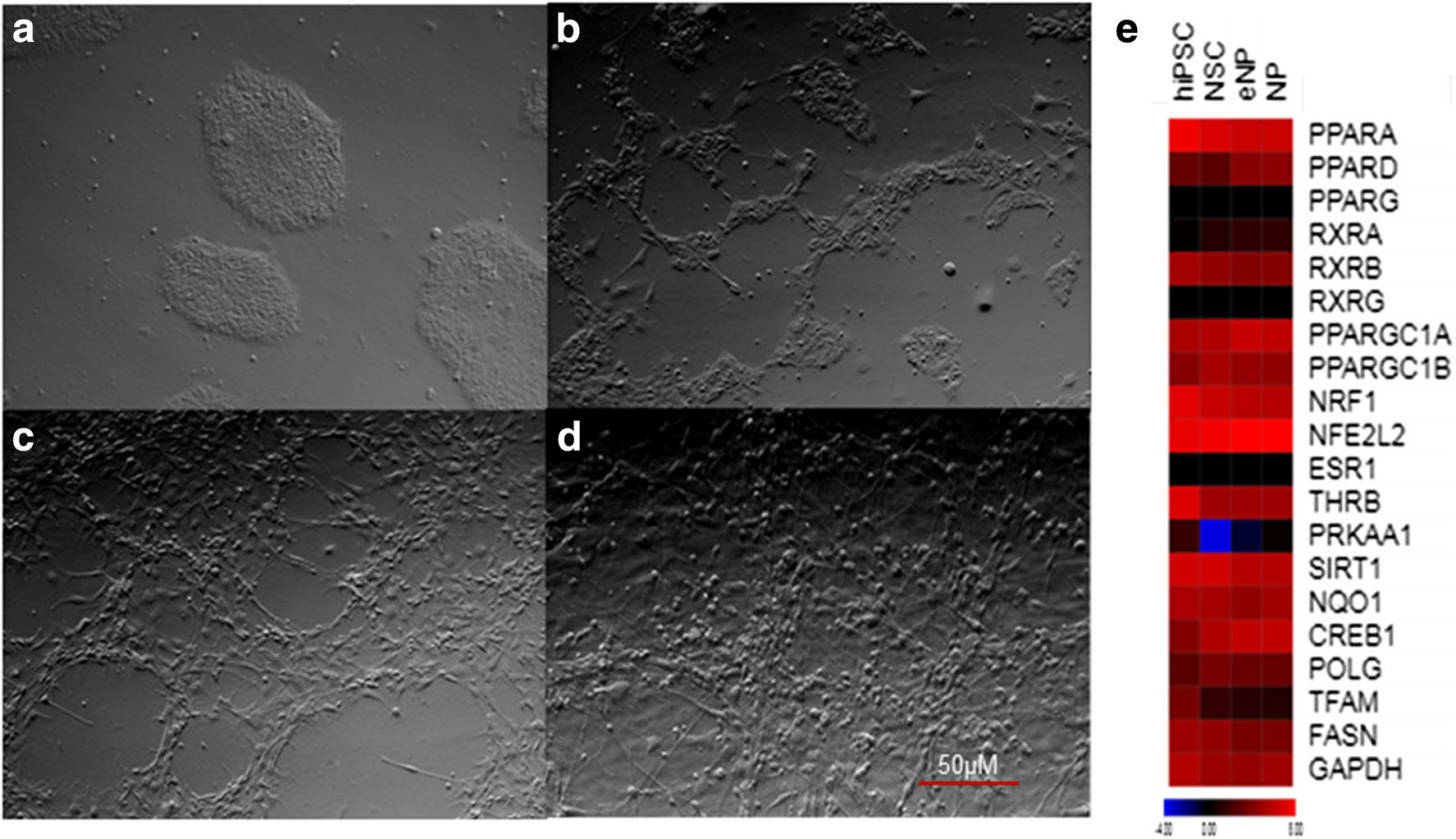
Comparison of the obtained cell populations at different stages of neural development. Phase-contrast images of cell morphology at iPSC (**a**), NSC (**b**), eNP (**c**), and NP (**d**). The heat map from RNA-Seq data (**e**) represents expression level of the selected genes involved in the PPARs and PGC1 alpha pathway at the hiPSC, NSC, eNP, and NP stages of development

The three stages of hiPSC neural differentiation: NSC (neural stem cells), eNP (early neural progenitors), and NP (neural progenitors), were obtained according to the protocol presented on Fig. 1. The morphology of these cells at different stages are presented on contrast-phase images (Fig. 2a–d). These lineage-related control cell populations obtained by our group and used in this study (Figs. 1 and 2a–d) have been fully characterized using RT-PCR, RNA-seq, and immunocytochemistry and these data have been recently published [11, 12].

### Expression of Selected Genes Involved in the Response to Bezafibrate

The expression of selected genes involved in the PPARs and PGC-1α pathways obtained with RNA-seq analysis are presented in the form of heat map on Fig. 2e. The panel of genes included *PPARA*, *PPARD*, *PPARG*, *RXRA*, *RXRB*, *RXRG*, *PPARGC1A*, *PPARGC1B*, *NRF1*, *NFE2L*2, *ESR1*, *THRB*, *PRKAA1*, *SIRT1*, *NQO1*, *CREB1*, *POLG*, *TFAM*, and *FASN*.

Out of the set of analyzed genes, only the expression of *PPARGC1A* gene, which encodes PGC-1α, the main regulator of mitochondrial biogenesis, revealed the significant difference (FC > 2; *p* < 0.0001) between compared populations (NSC, eNP, NP vs. hiPSC and eNP, NP vs. NSC). The supplementary data (Supplementary Data 1) show the statistical evaluation of the comparison of expression of genes presented on the heat map (Fig. 2e) between analyzed cell populations.

### Cell Viability

**Fig. 3:**
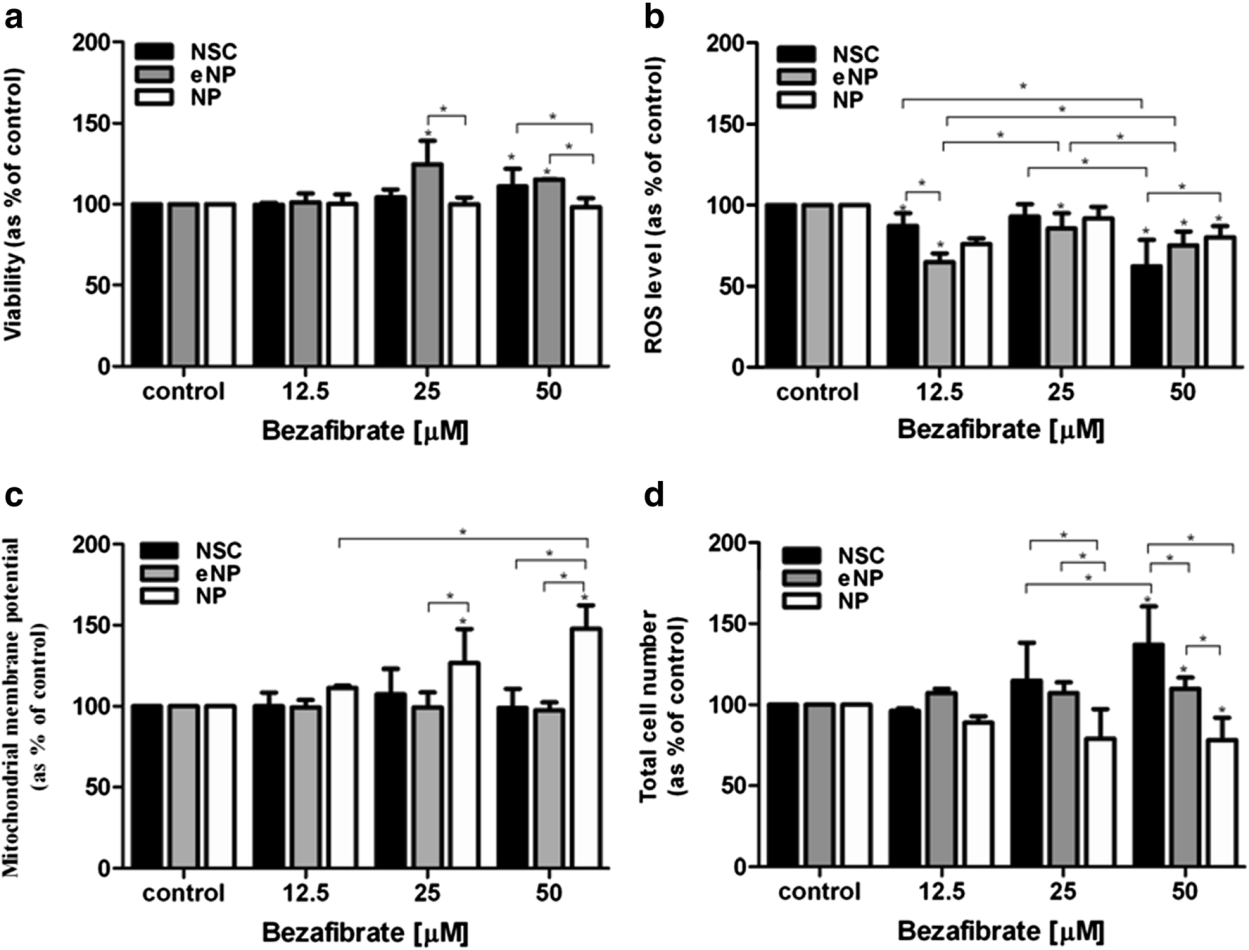
**a** Determination of viability (Alamar blue assay). **b** ROS level measured with DCFDA test. **c** Analysis of mitochondrial membrane potential (Mitotracker® Red CMXRos). **d** Total cell number (Janus green staining) in NSC, eNP, and NP cells after 5 days exposition to the various concentrations of BZ (0 μM, 12.5 μM, 25 μM, and 50 μM). The statistical significance (*), *p* < 0.05, between samples vs. control was obtained with one-way ANOVA with Tukey’s post-test. The statistical significance (*), *p* < 0.05, between analyzed groups was obtained with two-way ANOVA with Bonferroni’s post-test. Results shown in the brackets were obtained as mean with (± SEM) and present as [%] of control. Data (Fig. 3a–c) were normalized to total cell number obtained with Janus green staining (Abcam) according to the manufacturer’s protocol

12.5 μM concentration of BZ did not have a significant effect on cell viability in all tested populations (Fig. 3a). A significant increase in viability (in relation to control) was recorded for the eNP stage of development at dose 25 μM (124.77% (± 10.21). The highest tested dose of BZ (50 μM) significantly increased viability of NSC (111.03% (± 9.87) and eNP (115.31% (± 0.03) (with regard to control). We did not observe significant changes in viability of NP cells exposed to BZ. For 25 μM, we noted significant difference in viability after exposition to BZ between eNP and NP, while for 50 μM between NSC and NP as well as eNP and NP. The results are shown in Fig. 3a

**Fig. 4:**
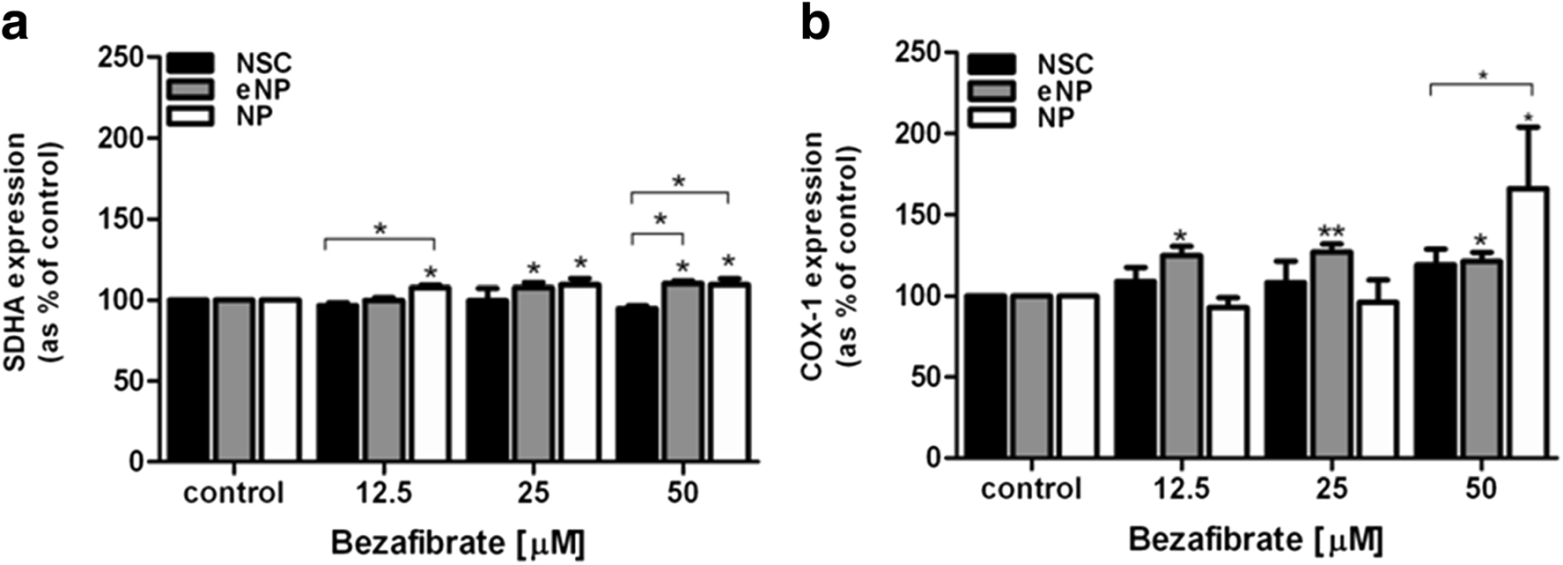
SDHA and COX-1 level at NSC, eNP, and NP cells treated with BZ. Mitochondrial biogenesis was evaluated by analysis of **a** succinate dehydrogenase complex flavoprotein subunit A (SDHA) protein level and **b** cyclooxygenase isoenzyme (COX-1) protein level. Brackets show statistical significance between samples vs. control (one-way ANOVA, Tukey’s post-test) and comparison between groups (two-way ANOVA, Bonferroni’s post-test): (*), *p* < 0.05. Results shown in the brackets were obtained as mean (± SEM) and presented as [%] of control. Data (**a**, **b**) were normalized to the cell number with Janus green staining (Abcam) according to the manufacturer’s protocol

### ROS Level

BZ reduced ROS level significantly (with regard to control) in all tested populations (NSC, eNP, and NP). In the eNP population, this decrease was significant at all tested doses: 12.5 μM (64.78% (± 5.33), 25 μM (85.61% (± 9.26), and 50 μM (75.12% (± 8.46). At the NSC stage of differentiation, significant reduction of the level of free radicals was recorded for doses 12.5 μM (87.20% (± 7.70)) and 50 μM (62.30% (± 16.22)). The strongest significant effect of BZ on ROS level in all tested populations was observed for dose 50 μM: NSC (62.30% (16.22)); eNP (75.17% (8.46)); NP (80.14% (± 6.96)). A significant difference between populations was noted only at dose 50 μM between NSC and NP. The data are shown in Fig. 3b.

### Mitochondrial Membrane Potential

BZ did not significantly change (relative to control) mitochondrial membrane potential (ΔΨm) in the population of NSC and eNP in any tested dose. A significant increase of ΔΨm was observed in the NP stage for doses 25 μM (126.62% (± 17.13)) and 50 μM (147.68% (± 10.35), but not for 12.5 μM (111.35% (± 1.01)). For 25 μM dose of BZ, a significant difference between eNP and NP cells was observed, while at 50 μM, NP vs. NSC and NP vs. eNP were significantly different (Fig. 3c).

### Total Cell Number

We showed a significant influence of BZ on the total cell number (relative to control) in all tested cell populations only at the dose 50 μM; while in NSC and eNP, the total cell number was increased significantly (136.87% (± 20.50) and 110.00% (± 6.37) respectively), at the NP stage of development, the response to BZ was not significant (78.22% (± 12.41)). When different stages of development were compared, significant differences were observed for the 25 μM and 50 μM dose, and NSC revealed the highest increase in total cell number. At 25 μM, a significant difference was observed between NSC and NP and also between eNP and NP, while at 50 μM among NP vs. NSC; eNP vs. NSC; and NP vs. eNP (Fig. 3d).

### Mitochondrial Biogenesis Evaluated at the Protein Level: SDHA and COX1

We observed a significant increase of SDHA protein level in NP cells treated with BZ vs. untreated control for all tested concentrations: 12.5 μM (107.59 (± 2.40)); 25 μM (109.49 (± 3.80)); 50 μM (109.52 (± 3.50)). BZ significantly enhanced the SDHA protein level in the eNP cells at doses 25 μM (107.61 (± 6.05)) and 50 μM (110.16 (± 1.57)). At the NSC stage of differentiation, BZ did not significantly affect the SDHA level in any of the tested concentrations. A significant difference: NP vs. NSC, was shown for 12.5 μM and 50 μM BZ. The difference between NSC and eNP was significant at dose 50 μM (Fig. 4a).

**Fig. 5:**
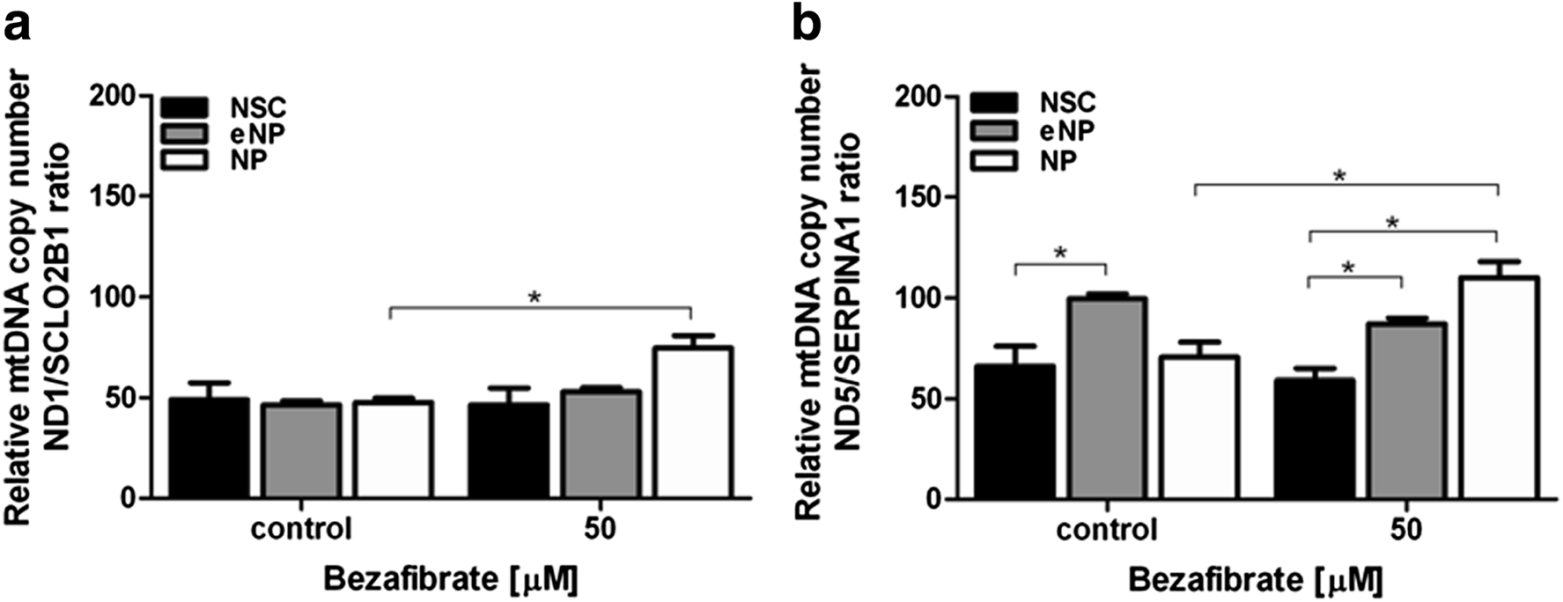
The relative mtDNA copy number was evaluated by the ratio of **a** *ND1* to *SCLO2B1*, and **b** *ND5* to *SERPINA1* in NSC, eNP, and NP cell populations exposed to 5 days of BZ (50 μM). Brackets show statistical significance between samples vs. control (Student’s *t*) and comparison between groups (two-way ANOVA, Bonferroni’s post-test): (*), *p* < 0.05. Results shown in the brackets were obtained as mean (± SEM) and are presented as [%] of control

BZ significantly increased the COX-1 protein level in the eNP at all tested concentrations: 12.5 μM (124.79 (± 11.39)); 25 μM (127.02 (± 11.11)); 50 μM (121.24 (± 12.44), and in the NP at 50 μM (166.18 (± 37.96)). A significant difference between tested groups was detected between NSC and NP (Fig. 4b).

### Relative mtDNA Copy Number

Relative mtDNA copy number was calculated as the (A) *ND1/SCLO2B1* ratio and (B) *ND5/SERPINA1* ratio, where *ND1* and *ND5* are encoded by mitochondrial genome and *SCLO2B1* and *SERPINA1* are encoded by nuclear genome. BZ significantly increased the ratio of *ND1/SCLO2B1* from 47.67 (± 3.09) to 74.50 (± 10.71) in NP stage. Relative mtDNA copy number in the NSC, eNP, and NP populations treated with BZ did not differ significantly between the tested groups (Fig. 5a).

**Fig. 6:**
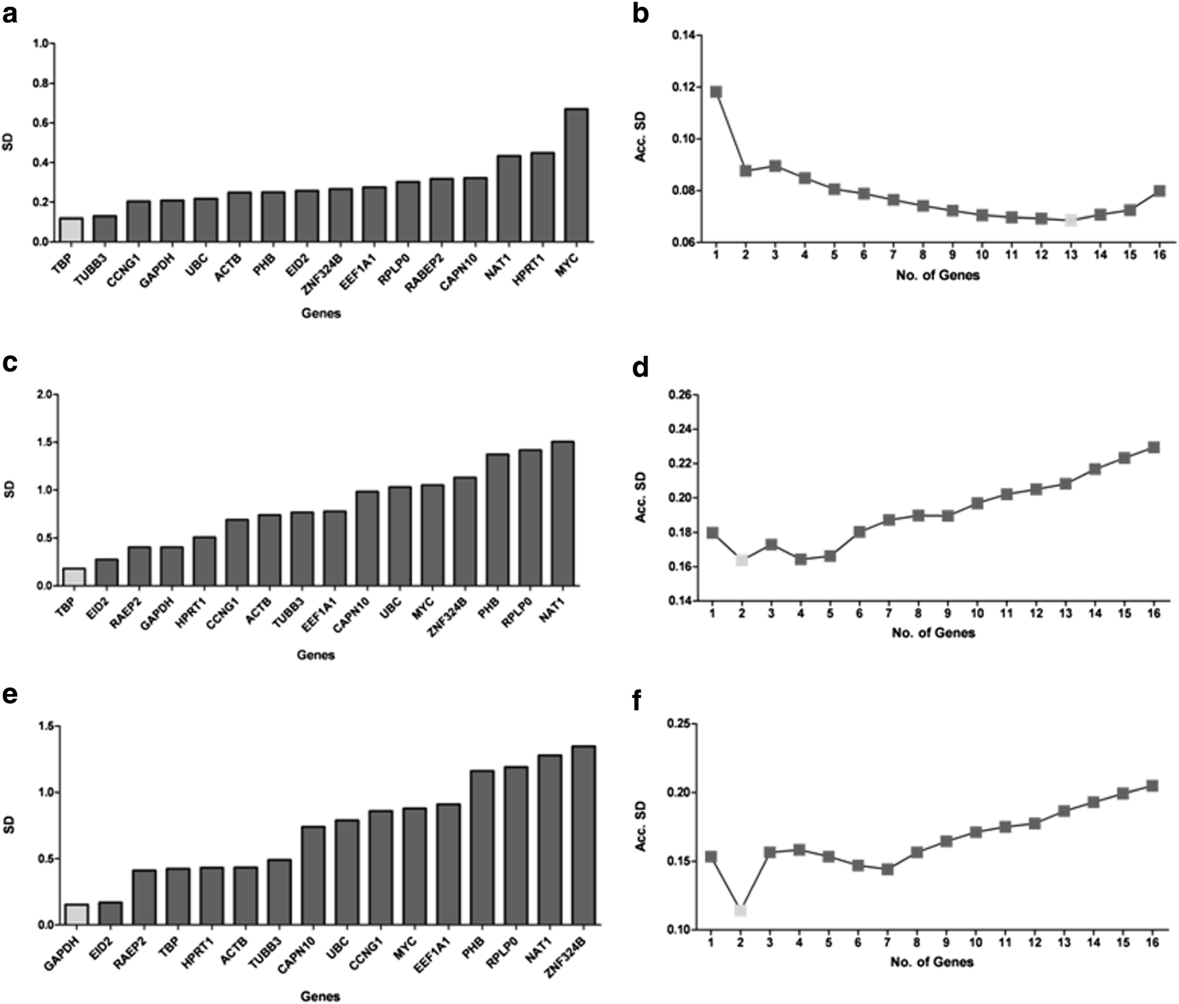
Estimation of the expression stability of 16 reference genes for NSC, eNP, and NP treatment with BZ (50 μM) and untreated control. All genes tested were ranked on the *x*-axis according to their expression stability, calculated with the NormFinder algorithm. The results are shown as SD of gene expression stability (**a**, **c**, **e**). The number of tested, potential reference genes is shown on the *x*-axis (**b**, **d**, **f**). Minimal value of the accumulated standard deviation (Acc. S.D) indicates the number of genes required for proper normalization in NSC (**b**) cells, eNP (**d**) cells, and NP (**f**)

BZ significantly enhanced the relative mtDNA copy number measured as the *ND5/SERPINA1* ratio only in the NP stage of differentiation from 70.50 (± 7.50) to 110.00 (± 8.00), when control to treated cells were compared. Comparison of starting, untreated cell populations revealed significant difference: NSC vs. eNP. BZ-treated populations were significantly different for eNP vs. NSC and NP vs. NSC (Fig. 5b).

### Validation of Reference Genes for qRT-PCR

**Fig. 7:**
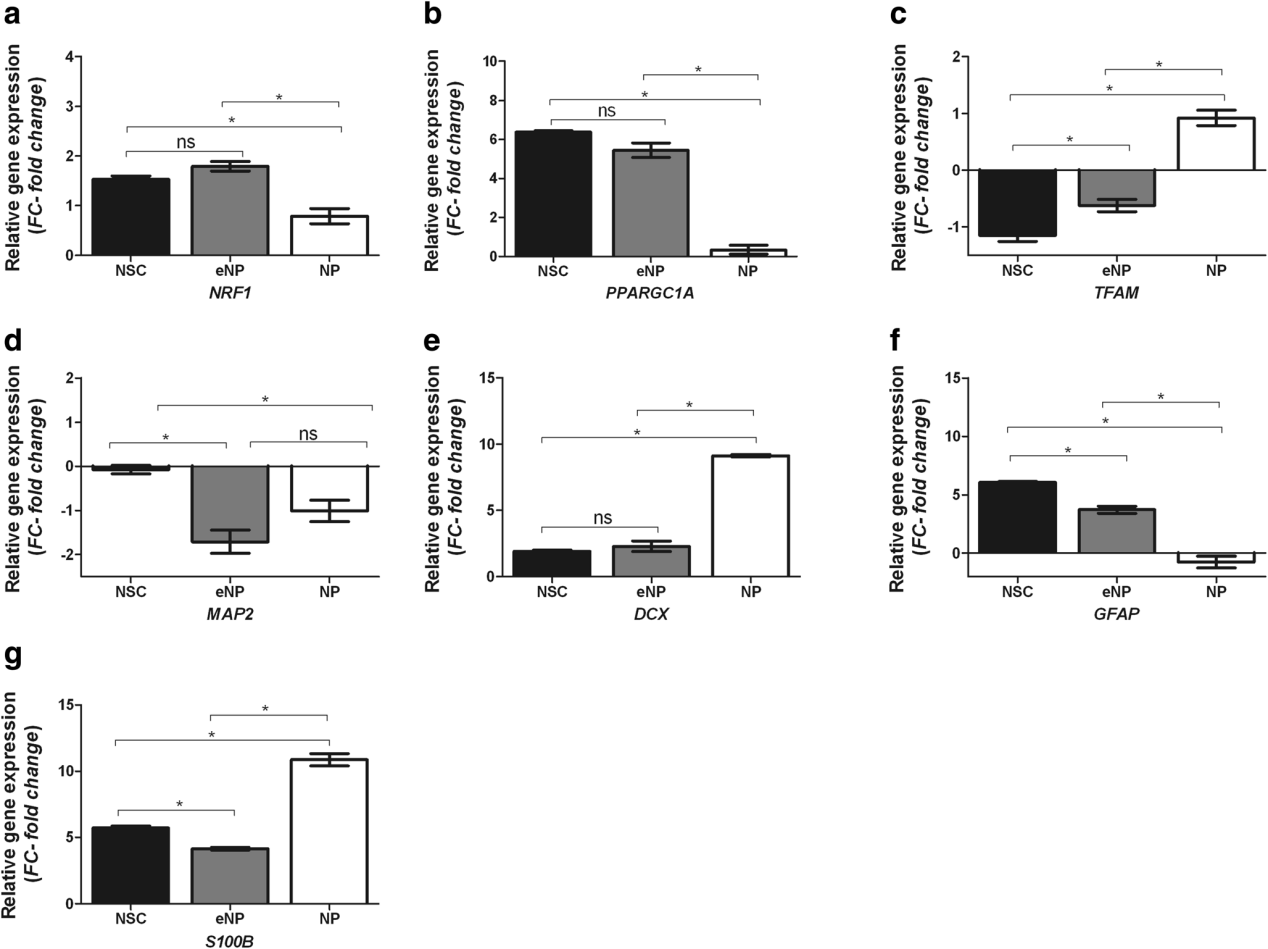
Relative gene expression (fold of changes vs. control) was determined in NSC, eNP, and NP cells after 5 days exposition to BZ (50 μM) by qRT-PCR methods. Relative gene expression was evaluated for key genes connected with regulation of mitochondrial biogenesis: **a** *NRF1*, **b** *PPARGC1A*, and **c** *TFAM*; neuronal: **d** *MAP2* and **e** *DCX*; and astrocytic: **e** *GFAP* and **f** *S100B* differentiation. Results presented in the brackets are represented as the mean value (± SEM). Brackets show statistically significant differences between NSC, eNP, and NP obtained with one-way ANOVA with Tukey’s post-test: (*), *p* < 0.05

NormFinder was used to rank the tested genes according to their average expression stability value from the most stable expression (lowest SD value) to the least stable expression (highest SD value). The ranking of the 16 potential housekeeping genes according to their expression stability values is shown in Fig. 6a, c, e. The lowest values correspond to the most stable gene expression. To obtain the optimal number of housekeeping genes for normalization in RT-qPCR relative gene expression in NSC cells (Fig. 6b), eNP cells (Fig. 7d) and NP cells (Fig. 6f), accumulated standard deviation (Acc. S.D.) calculation was performed. Thirteen genes (*TBP*, *TUBB3*, *CCNG1*, *GAPDH*, *UBC*, *ACTB*, *PHB*, *EID2*, *ZNF324B*, *EEF1A1*, *RPLP0*, *RABEP2*, and *CAPN10*) were selected as reference genes for the NSC (Fig. 6b). For the NP and eNP, two reference genes were predicted by NormFinder program. These are *TBP* and *EID2* or *GAPDH* and *EID2*, respectively.

### Relative Gene Expression Involved in Regulation Mitochondrial Biogenesis and Neural Differentiation

Expression of the key genes controlling mitochondrial biogenesis: *NRF1*, *TFAM*, and *PPARGC1A*, and genes involved in neural (*DCX*, *MAP2*) and glial (*GFAP*, *S100B*) differentiation was tested using qRT-qPCR. BZ exposition results with upregulation of *NRF1* gene expression at all tested neural stages of development. *NRF1* mRNA expression level was enhanced; about 2-fold at the stage of early neural progenitors (1.79 (± 0.22)), about 1.5-fold at NSC (1.53 (± 0.15)), and about 1-fold at NP (0.79 (± 0.34)). A significant difference of relative *NRF1* gene expression was detected: NP vs. NSC and NP vs. eNP (Fig. 7a).

*PPARGC1A* expression level was increased about 6-fold in NSC exposed to BZ (50 μM) compared to the untreated control. In eNP and NP, upregulation of *PPARGC1A* gene expression about 5-fold in eNP (5.45 (± 0.84)) and about 0.3-fold in NP (0.35 (± 0.32)) was observed. Significant difference in *PPARGC1A* gene expression was noted: NP vs. NSC and NP vs. eNP, but not eNP vs. NSC (Fig. 7b).

*TFAM* expression level after BZ treatment was different at specific stages of neural development. It was downregulated in the NSC (− 1.16 (± 0.22)) and eNP (− 0.625 (± 0.244)) population while in NP (0.92 (± 0.30)), upregulation was observed. A significant difference was detected: eNP vs. NSC; NP vs. NSC; and NP vs. eNP (Fig. 7c).

*MAP2* was downregulated upon BZ treatment at all tested populations; however, the strongest downregulation was observed in the eNP (− 1.71 (± 0.59)) and in the NP (− 1.01 (± 0.54)) stages. At NSC stage of development, this downregulation was not significant (0.08 (± 0.21)). When the obtained data were compared between different stages, significant difference between *MAP2* relative gene expression was observed in NP vs. NSC and eNP vs. NSC (Fig. 7d).

*DCX* gene expression was upregulated in all population after treatment in BZ, for eNP (2.28 (± 1.21)) and for NSC (1.91 (± 0.28)). The strongest effect of BZ exposition for *DCX* expression was observed in the NP cells (± 9.14 (0.39)). The difference in relative gene expression of DCX as the response to BZ was observed between NP vs. NSC and NP vs. eNP, but not between eNP vs. NSC. (Fig. 7e).

The *GFAP* mRNA expression level was enhanced by BZ (50 μM) treatment about 6-fold (6.06 (± 0.20)) in NSC and about 4-fold (3.72 (± 0.68)) in eNP, while was not downregulated at NP (− 1.94 (± 0.39)) as compared to untreated control. Upon BZ treatment, *GFAP* relative gene expression was different at tested stages of development. Significant differences were noted between relative *GFAP* expression in the eNP vs. NSC and NP vs. eNP (Fig. 7f).

The *S100B* gene expression was increased significantly after exposition to BZ in all tested populations: NSC (5.73 (± 0.570)); eNP (4.15 (± 0.295)); and NP (10.89 (± 1.83)) (Fig. 7g). The strongest upregulation of *S100B* gene was in the NP population. *S100B* relative gene expression level after BZ exposition was different at specific stages of neural development eNP vs. NSC; NP vs. NSC; and NP vs. eNP.

The summary of the results (presented in Table 3) indicated that only at the NP stage of differentiation all the parameters indicating mitochondrial biogenesis were upregulated upon stimulation by BZ.

**Table 3:** Summary of the influence of bezafibrate (50 μM) on NSC, eNP, and NP

		NSC	eNP	NP
Viability		↑ (*)	↑ (*)	↓ (ns)
ROS level		↓ (*)	↓ (*)	↓ (*)
Mitochondrial membrane potential		↓ (ns)	↓ (ns)	↑ (*)
Total cell number		↑ (*)	↑ (*)	↓ (*)
Protein expression	SDHA	↓ (ns)	↑ (*)	↑ (*)
	COX-1	↑ (ns)	↑ (*)	↑ (*)
mtDNA copy number	*ND1/SCLO2B1* ratio	↓ (ns)	↑ (ns)	↑ (*)
	*ND5/SERPINA1* ratio	↓ (ns)	↓ (ns)	↑ (*)
Gene expression (fold of change)	*NRF1*	↑ (1.53)	↑ (1.79)	↑ (0.79)
	*TFAM*	↓ (− 1.16)	↓ (− 0.63)	↑ (0.92)
	*PPARGC1A*	↑ (6.39)	↑ (5.45)	↑ (0.35)
	*MAP2*	↓ (− 0.08)	↓ (−1.71)	↓ (− 1.01)
	*DCX*	↑ (1.91)	↑ (2.28)	↑ (9.137)
	*GFAP*	↑ (6.06)	↑ (3.72)	↓ (− 1.94)
	*S100B*	↑ (5.73)	↑ (4.15)	↑ (10.89)

*NSC* neural stem cells, *eNP* early neural progenitors, *NP* late neural progenitors, *ns* not significant, ↑ upregulation, ↓ downregulation

*p < 0.05

### Analysis In Silico by Genemania

**Fig. 8:**
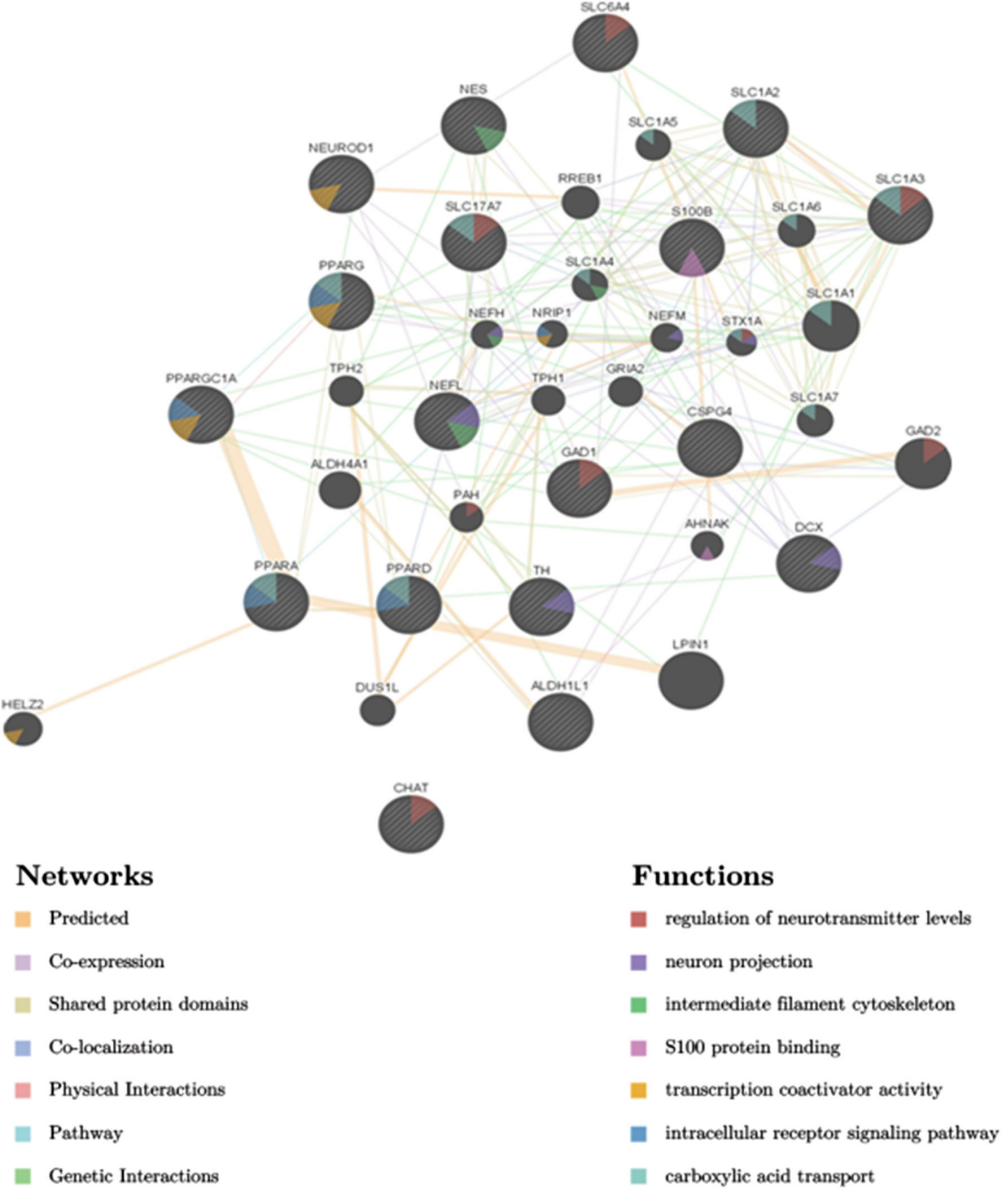
Modeling *in silico* the interaction between selected genes involved in PGC-1α pathway and neuronal differentiation. The *in silico* analysis of function and interaction between PPAR receptors (*PPARA*, *PPARD*, *PPARG*), coactivator *PAPRGC1A* gene and neural (*NES*), neuronal (*DCX*, *NEFL*, *NEFM*, *NEFH*), neuronal subtypes (*TH*, *CHAT*, *SLC17A7*, SLC1A1, *GAD1*, *SLC6A4*), as well as astrocyte (*ALDH1L1*, *SLC1A3*, *SLC1A2*, *S100B*) and oligodendrocyte (*CSPG4*) markers was performed in the Genemania web tool (https://genemania.org/) to show the networks of interaction between the selected genes (cytoscape). The *in silico* evaluation present function of analyzed genes and network of interactions between them. Within the function category, analyzed genes were divided into the following groups: neuron projection (*NEFH*, *NEFM*, *BEFL*, *DCX*, *TH*); intermediate filament cytoskeleton (*NES*, *NEFH*, *NEFL*); intracellular receptor signaling pathway (*PPARA*, *PPARD*, *PAPRG*, *PPARGC1A*); S100B protein binding (*S100B*); regulation of neurotransmitter level (*CHAT*, *SLC17A7*, *SLC1A3*, *SLC6A4*, *GAD1*); carboxylic acid transport (*PPARA*, *PPARG*, *PAPRD*, *SLC1A3*, *SCL17A7*, *SLC1A1*, *SLC1A2*). Within the network category analyzed genes were divided, based on the type of interaction, to the following groups: physical interaction (1.41%), prediction (63.88%), genetic interaction (0.33%), shared protein domains (12.06%), co-localization (3.71%), co-expression (17.75%), and pathway (0.86%)

To determine the interrelationship between genes coding PPAR receptors (*PPARA*, *PPARD*, *PPARG*), *PAPRGCA1* and markers of neural cells (*NES*), neuronal cells (*DCX*, *NEFL*, *NEFM*, *NEFH*, *TH*, *CHAT*, *SLC17A7*, *SLC1A1*, *GAD*, *SLC6A4*), astrocytic markers (*ALDH1L1*, *SLC1A3*, *SLC1A2*, *S100B*), and oligodendrocyte marker (*CSPG4*), Genemania network analysis was applied, the results of which are displayed in Fig. 8. The derived network consisted of 22 of core genes and 15 additional genes that were pulled in by Genemania. In our research, we focused on gene expression studies. Genemania showed also the relationships in pathways, shared protein domains, co-localization, and co-expression between analyzed genes and their products. Detailed results are presented in Fig. 8.

## Discussion

In this report, we aimed to answer the question whether upregulation of mitochondrial biogenesis by BZ in hiPSC can be related to the regulation of their neural fate commitment.

The question addressed in this study was whether BZ can influence neural differentiation of hiPSC and whether the cellular response depends upon the stage of neural development. Since BZ is a well-known drug influencing mitochondrial biogenesis [4], we aimed to find out whether upregulation of mitochondrial biogenesis by BZ in hiPSC is developmental stage specific and can be linked to the regulation of their neural fate commitment. We investigated the cellular and molecular responses to BZ at three different stages of neural differentiation, covering the early developmental period: NSC, eNP, and NP obtained from human iPSC. It is important to note that NSC, eNP, and NP were shown to differ significantly in terms of expression of *NES*, *MAP2*, and *GFAP* [11]. The hiPSC-derived NSC, eNP, and NP model which we used in the BZ study was previously investigated by us in the PQQ and IDB study [11, 12]. All our experiments have shown developmental stage-dependent sensitivity of hiPSC in response to the factors involved in mitochondrial biogenesis (PQQ, IDB, and BZ); however, while PQQ and IDB treatment specifically enhanced mitochondrial biogenesis in eNP stage of development, BZ treatment was the most potent at NP stage.

Our results indicate that BZ enhances the expression of *PPARGC1A* gene in a stage-specific manner and it is not directly linked to its influence on mitochondrial biogenesis. The highest upregulation of *PPARGC1A* upon BZ treatment was observed at the NSC and eNP stage of differentiation and was accompanied by the upregulation of *GFAP*, similarly to other drugs (PQQ, IDB) tested previously by our group [11, 12]. Alongside other astrocytic markers, *S100B* was upregulated by BZ in all tested cell populations with the strongest effect in the NP stage. As mentioned above, the significant upregulation of mitochondrial biogenesis upon BZ treatment was also observed at NP stage of differentiation. Taken together, this data and from our previous studies may suggest that induction of mitochondrial biogenesis positively influence expression of astroglial markers. However, in case of BZ, and its influence on *GFAP* expression, such association does not exist. It is worth to note that in all our studies, upregulation of astrocytic markers coexists with upregulation of *PPARGC1A* and this is linked with downregulation of *MAP2* regardless of developmental stage-specific induction of mitochondrial biogenesis. Thus, our data suggest the key role of PGC-1α pathway in the regulation of the early neuronal and astrocytic fate commitment. The in silico analysis showed that main role of *PARGC1A* genes was related with involving in the PPAR receptor intracellular signaling pathway. *PPARGC1A* gene participates in the network connected with neural development but directly interacts only with the PPARA and PPARG genes. The interaction of *PPARAC1A* with other elements of the network should be deeply analyzed, since up to now it has not been sufficiently understand.

The correlation of astrocytic induction with PGC-1α expression was described previously in glioblastoma cells. Xing and colleagues [20] showed that PGC-1α-deficient glioblastoma cell line treated with N^6^,2′-dibutyryladenosine 3′5′-cyclic monophosphate (dbcAMP) was characterized by abrogated induction of *GFAP* expression, mtDNA content, and oxygen consumption rate (OCR), while overexpression of PGC-1α was liked to enhanced *GFAP* expression, mtDNA content, and OCR. Other studies indicated that stimulating mitochondrial biogenesis in mouse pluripotent stem cells (mESC) reduces pluripotency and favors cell differentiation or commitment to specific lineages [21]. Thus, we suspect that modulation of the specific pathway which controls mitochondrial function may stimulate commitment to specific neural lineages.

It was documented that adult mouse neural stem cells possess all three PPAR isotypes with nuclear localization which suggested their important role in process of neural differentiation as transcription factors [22]. However, the activity of these receptors has not been examined. To our knowledge, no literature data showing the expression and activity of PPAR receptors in the human NSC were reported.

Our RNA-seq data revealed significant upregulation of expression *PPARGC1A* in NSC, eNP, andNP in control populations as compared to hiPSC (Supplementary Table 1). The *PPARGC1A* gene is one of the most important factors which control mitochondrial biogenesis in the cells [23]. *TFAM* gene expression was downregulated during hiPSC neural differentiation [11]. The treatment with BZ reversed *TFAM* expression profile: the highest upregulation was observed in NP stage of differentiation, which is consistent with the induction of other markers of mitochondrial biogenesis upregulated upon BZ treatment only in NP stage.

Studies of other groups suggested also potential role of PPAR’s specific isotypes in the regulation of cell proliferation, death, and differentiation [22] but information on the impact of fibrates on the differentiation process is very limited. Data obtained by Cimini and colleagues [24] suggested that PPAR’s may be involved in the neural stem cell (NSC) differentiation. The PPARα was shown to be involved in astrocytic differentiation [25] and the PPARβ/δ in the in vitro neuronal maturation [26]. Studies with neuroblastoma cell line demonstrated that PPARβ/δ agonists trigger neuronal differentiation [24]. It is also known that PPARγ are sensors capable of adapting gene expression to integrate various lipid signals; however, PPARα and PPARβ/δ are strongly involved into mitochondrial metabolism through fatty acid oxidation (FAO), thus have impact on mitochondrial metabolism and OXFOS [27].

Our RNA-seq data showed that the *PPARα* and *PPARβ/δ* but not *PPARγ* are expressed in all tested populations including starting population of hiPSC similar to the expression of *FASN*, the main gene involved in the regulation of fatty acid synthesis. Cimini and colleagues [22] identified a large lipid droplet in the cytoplasm of adult mouse NSC and suggested a role of PPARγ in the NSC differentiation into oligodendrocytes. Since the protocol of neural differentiation used in this study allows to get the cells with neuronal and astrocyte phenotypes, but not oligodendrocytes [11, 12], the lack of PPARγ expression is not surprising. The PPARγ is the important factor which controls adipocyte differentiation as well as in cellular types where lipogenesis occurs, such as oligodendrocytes [28, 29]. In our work, we concentrated mostly on the role of BZ on the *PPARGC1A* gene expression because earlier data obtained with RNA-seq methods showed the significant differences in the *PAPRGC1A* expression level between compared cell populations. We have also proved that upregulation of *PPARGC1A* gene by BZ coexists with *GFAP* early astrocytic and *S100B* more advanced astrocytic marker upregulation. While GFAP is also the marker of NSC (radial glial cells), it was documented that during development, enhanced S100B expression defines a state in which GFAP-expressing cells lose their neural stem cell potential [30] and acquire a more mature developmental stage. This nicely correlates with downregulation upon BZ treatment of the advanced neuronal marker *MAP2* and the same time upregulation of the *DCX*, defining the early neuronal marker. Such data strongly suggest that *PAPRGC1A* plays important role in the mitochondrial biogenesis regulation but also may act independently in the control of neural fate decision.

**Fig. 9:**
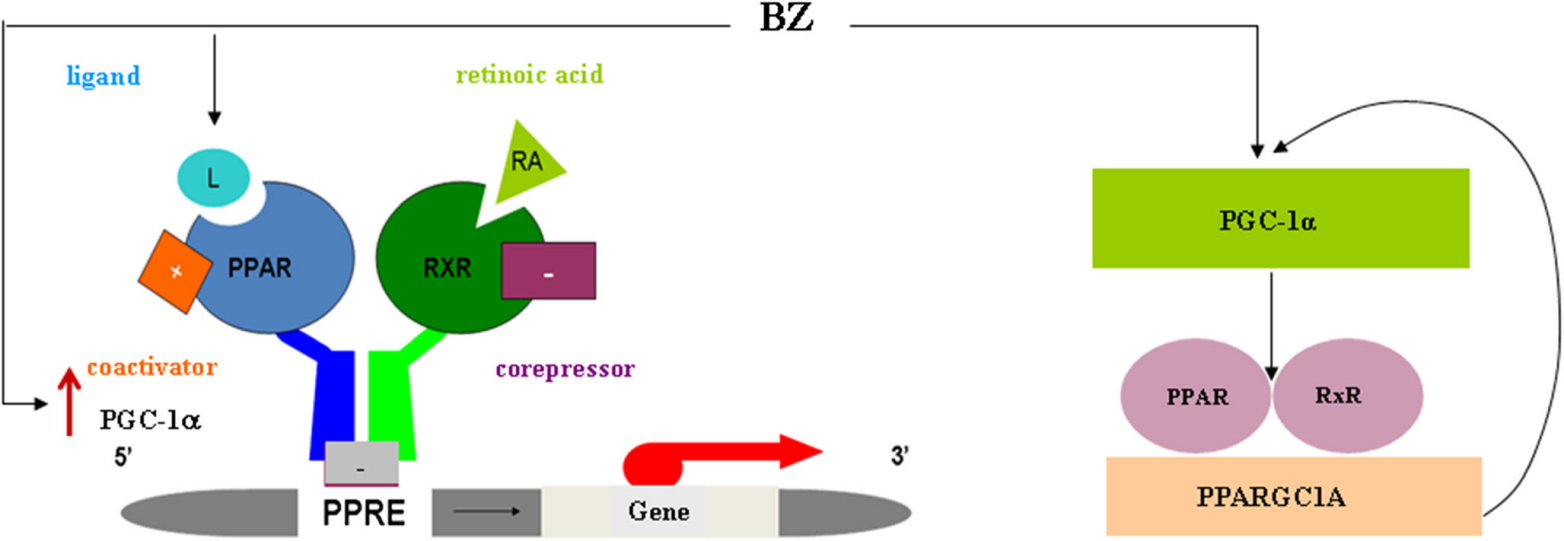
The role of BZ in the PPARA/RXRA ligand-dependent (PPARAGC1A) gene transcription regulation. As a transcription factor, peroxisome proliferator-activated receptor alpha (PPAR-alpha) encoded by the PPARA gene regulates transcription of PPAR response element-containing promoter genes after heterodimerization with retinoid X receptor alpha (RXRA). The complex of PPARA/RXRA binds to specific regions on the DNA of target genes. The DNA consensus sequence of peroxisome proliferator hormone response elements (PPRE) is AGGTCANAGGTCA, with N being any nucleotide. The function of PPAR-alpha is modulated by ligands and by a number of coactivator and corepressor proteins. Upon PPARA/RXRA complex binding into the ligand-binding domain, transcription of target genes is up- or downregulated. Negative regulation of PPARA/RXR transcription activity is related to recruitment of the transcriptional corepressors (for example, NCoR and SMRT), while positive activation is related to recruitment inter alia PGC1α coactivator encoded by PPARGC1A gene. In this molecular mechanism, retinoic acid plays a role of the major selective agonist of the endogenous mammalian RXR receptor, while BZ plays a role of the ligand that binds to a PPAR-α receptor. BZ as an agonist of PPARα upregulates activity of this receptor and induces *PPARGC1A* gene transcription, which in positive feedback mechanism further stimulates the system. BZ can upregulate PPARGC1A transcription on dependent and independent pathways from PPARA/RXRA complex. See text for the references

The mechanism of possible interaction of BZ with PGC-1α pathway is presented in Fig. 9. BZ acts as the agonist/ligand binding to a PPARα receptor, but also can interact with PGC-1α protein, upregulating *PPARGC1A* (encoding PGC-1α) transcription by independent pathway from PPARA/RXRA complex [31]. The peroxisome proliferator-activated receptors (PPARs) when activated by BZ form heterodimers with the retinoid X receptor (RXR) and after heterodimerization, the complex of PPARA/RXRA bind to specific regions on the DNA of target genes [32] through a PPRE—response element, which is also present in the PGC-1α gene promoter. Increased PGC-1α expression reinforces PPAR activation, therefore triggering a positive autoregulatory loop of the PPAR-PGC-1α axis. The PPARs function as obligate heterodimers with RXR. PPARα, PPARβ/δ, and PPARγ are encoded separately but all of them take part in the regulation of important metabolic pathways [33]. Negative regulation of PPARA/RXR transcription activity is related to recruitment the transcriptional corepressors, for example, NCoR and SMRT, while positive activation is related to recruitment inter alia PGC1-alpha coactivator encoded by PPARGC1A gene [34, 35]. We must emphasize that transcription activity of *PAPRs* during neural differentiation and their role in the neural fate commitment is poorly understood. During hiPSC neural differentiation *RXRA*, *RXRB* was expressed in all tested cell types while *RXRG* was not. The changes in the expression of PPARs and RXRs upon BZ treatment have not been investigated in this study. However, the differences observed by us in the expression of *PPARGC1A* gene after BZ treatment are strongly suggesting the involvement of this pathway in activation of mitochondrial biogenesis and fate commitment switch observed in this study.

We have confirmed antioxidant capacity of BZ, revealed by a significant decrease of the ROS level and the upregulation of *NRF1* gene expression in all tested cell populations [36, 37]. NRF1 is one of the key transcription factors which regulates metabolic and antioxidant genes and modulates mitochondrial DNA transcription and replication [38]. We have shown that BZ treatment upregulates both *PPARGC1A* and *NRF1* during neural differentiation of hiPSC. Documented interference of those two factors is crucial to antioxidant defense and redox balance. Reduction of ROS in NP stage was accompanied by increase in the mitochondrial membrane potential and this relation is typical for other cellular systems [39, 40].

Resting mitochondrial membrane potential was previously linked to the differentiation potential of mESC [41]. mESC with low resting mitochondrial membrane potential had great propensity for in vitro mesodermal differentiation but did not efficiently form teratomas in vivo, whereas mESC with high resting mitochondrial membrane potential favored multi-lineage differentiation. These examples suggest that modulation of mitochondrial membrane potential can influence the cell fate decision. In our study, we have observed downregulation of mature neuron *MAP2* and early astrocytic *GFAP* marker and the same time strong upregulation of early neuronal marker *DCX* and mature astrocytic *S100B* in NP stage in relation to the significant increase of mitochondrial membrane potential induced by BZ. The role of increased of mitochondrial membrane potential upon BZ treatment at NP in the induction of mitochondrial biogenesis is still to be elucidated.

One of the main goals of mitochondrial disease therapy is to improve mitochondrial function by increasing the number of mitochondria [42]. We demonstrated that the treatment with BZ increased mitochondrial biogenesis by all tested factors (significant upregulation of SDHA and COX1 protein expression, *NRF1*, *TFAM*, *PPARGC1A* gene expression, and increased mtDNA copy number only in NP stage of hiPSC neural differentiation). This was not related to the cell viability, which was not changed by BZ at this stage of development.

The expression of two proteins important for mitochondrial function: SDHA and COX-1, was measured as one of the indicators of mitochondrial biogenesis. SDHA is major catalytic subunit of succinate-ubiquinone oxidoreductase [43], a complex of the mitochondrial respiratory chain, while COX1 is the component of the respiratory chain that catalyzes the reduction of oxygen to water [44]. We have shown a significant increase of SDHA and COX-1 expression upon BZ treatment in eNP and NP stages, which may indicate a positive drug effect on mitochondria functionality. The level of mtDNA copy number was the second indicator of mitochondrial biogenesis. We detected a significant increase of mtDNA copy number upon BZ treatment but only at the NP stage of development.

In addition, our data indicate that only at NP stage the upregulation of *TFAM* expression after BZ treatment is concomitant with an increase in mitochondrial DNA copy number. The developmental stage-specific relation of the increase in *TFAM* expression and mtDNA copy level was also demonstrated after exposition to PQQ and IDB but in these cases at the eNP stage of development [11, 12]. In conclusion, we have shown that regulation of mitochondrial biogenesis by *TFAM* in hiPSC is modified by selected drugs in neural stage development-dependent manner.

Through the recent studies of our group, we have observed an interesting molecular relationship of the expression of selected genes important for mitochondrial biogenesis with the expression of genes typical for neuronal and astrocytic differentiation. In that respect, the most important result in our study seems to be the upregulation of the expression of *PPARGC1A*-encoded coactivator PPARs-PGC-1α, which is known to activate NRF1/2, PPAR, YY1, and ERR transcription factors, controlling key mitochondrial genes (OXPHOS subunits, Krebs cycle enzymes, fatty acid β-oxidation, proteins involved in mitochondrial protein import and assembly) [45]. The highest upregulation of *PPARGC1A* upon BZ treatment was observed at the NSC and eNP stage of differentiation and as mentioned above accompanied by upregulation of *GFAP* and *S100B*. *GFAP* gene expression was confirmed also in the case of other drugs tested by our group (PQQ, IDB) [11, 12].

Surprisingly in this study, the increase of mitochondria biogenesis measured by all tested factors (protein expression, mtDNA copy number, and expression of selected genes, linked to mitochondria function) for the NP stage of differentiation was accompanied with upregulation of early neuronal *DCX* and mature astrocyte *S100B* markers. Induction of mitochondrial biogenesis, dependent upon the stage of hiPSC neural differentiation, was shown by our group in the case of PQQ [11] and IDB [12], but only at eNP, not at NSC and NP stages of development and this coexists with astrocytic fate commitment.

Further studies are needed to explain the molecular mechanisms of developmental stage-specific induction of mitochondrial biogenesis and the neural fate specification. We think that the defined cellular “metabolic state” is necessary to induce mitochondrial biogenesis; therefore, stimulation of mitochondrial biogenesis was not observed at the NSC stage exposed to PQQ [11] and IDB [12] as well as BZ in this study. However, BZ was efficient at the more advanced stage of differentiation (NP) than other drugs.

One of the explanations is that the mechanisms of action of PQQ [46] and IDB [47] are antioxidant-related, while BZ regulates fatty acid metabolism. IDB and PQQ belong to an antioxidant group, while BZ impact on mitochondria is related to its caloric restriction functions [48]. PQQ [11] and IDB [12] induced the strongest mitochondrial biogenesis at the stage eNP, while BZ at the stage of NP. One of the potential reasons that BZ is effective in NP stage to induce mitochondrial biogenesis can be a fact that proliferating neural progenitors at this stage contain high levels of fatty acid synthase [49]. Activation of acetyl-CoA (Ac-CoA) carboxylase (ACC) and fatty acid synthase (FASN) at neural progenitors increase fatty acid synthesis from Ac-CoA to fuel phospholipid membrane synthesis [50].

In conclusion, in our study, the expression of receptor *PPARα* is on the highest level as compared to other PPARs in all tested cell populations. *PPARG* and *RXRG* play potentially important role in the oligodendrocyte differentiation. Lack of expression of these receptors coexists with lack of oligodendrocytic phenotypes. Our results indicated that upon BZ treatment, modification of neural cell fate decision is linked to the induction of mitochondrial biogenesis (upregulation of *S100B* and downregulation of *MAP2* at NP stage) but not for all tested differentiation markers (e.g., *GFAP*). For the differentiation switch, PGC-1α and PPAR signaling pathway seems to play the crucial role: elevated expression of astroglial markers *GFAP* and *S100B* and downregulation of *MAP2* always coexist with upregulation of *PPARGC1A*. Thus, the possible way of action of BZ in activation of astrocytogenesis is through the pathway dependent from PGC-1α and PPARα. One of the most important results in our study seems to be the upregulation of the expression of all tested genes as well as protein markers crucial for mitochondria function only in NP stage of development. Thus, we have confirmed that the positive effect of BZ on mitochondrial biogenesis is dependent on neural stem cell stage of differentiation. However, it is suggested that for the control of neural fate decision, the mutual interplay between mitochondrial biogenesis, PPAR receptor activity, and maturity of mitochondria are required. Our study indicates a new potential use of BZ as the stimulant of mitochondrial biogenesis in neural progenitors. This may be important for the development of pharmacotherapy of neural diseases with mitochondrial dysfunction. To the best of our knowledge, we provided the first study investigating BZ role in neural fate commitment modulation.

## Electronic Supplementary Material

ESM 1 Comparison gene expression (RNA-seq) involved in mitochondrial biogenesis, e.g. regulated by PPAR’s receptors or PGC-1α coactivator (FC>2, p<0,0001) at the obtained cell populations at different stages neural of development. (XLSX 27 kb)

ESM 2 (PDF 429 kb)

## Abbreviations

ACTB: Actin beta
bFGF: Basic fibroblast growth factor
BZ: Bezafibrate
CAPN10: Calpain 10, calcium-activated neutral proteinase 10
CCNG1: Cyclin G1
CHAT: Choline O-acetyltransferase
MT-CO1: Mitochondrially encoded cytochrome c oxidase I (COX-1)
CREB1: cAMP-responsive element-binding protein 1
DCFHDA: Dichloro-dihydro-fluorescein diacetate
CSPG4: Chondroitin sulfate proteoglycan 4
DCX: Doublecortin
EEF1A1: Eukaryotic translation elongation factor 1 alpha 1
EGF: Epidermal growth factor
EID2: EP300-interacting inhibitor of differentiation 2
eNP: Early neural progenitors
ERRs: Estrogen-related receptors
ESR1: Estrogen receptor 1
ETC: Electron transport chain
FAO: Fatty acid oxidation
FASN: Fatty acid synthase
GAD1: Glutamate decarboxylase 1
GAPDH: Glyceraldehyde-3-phosphate dehydrogenase
GFAP: Glial fibrillary acidic protein
hESC: Human embryonic stem cells
hiPSC: Human-induced pluripotent stem cells
HPRT1: Hypoxanthine phosphoribosyltransferase 1
IDB: Idebenone
Ki67: Proliferation marker protein Ki-67
MAP 2: Microtubule-associated protein 2
mESC: Mouse embryonic stem cells
MYC-V: Myc avian myelocytomatosis viral oncogene homolog
NANOG: Homeobox protein NANOG
NAT1: *N*-Acetyltransferase 1
NCOR1: Nuclear receptor corepressor 1, thyroid hormone- and retinoic acid receptor-associated corepressor 1
ND1: Mitochondrially encoded NADH-ubiquinone oxidoreductase core subunit 1 (mt-ND1)
ND5: Mitochondrial membrane respiratory chain NADH-ubiquinone oxidoreductase chain 5 (mt-ND5)
NEFL: Neurofilament, light polypeptide
NEFM: Neurofilament, medium polypeptide
NEFH: Neurofilament, heavy polypeptide
NES: Nestin
NEUROD1: Neuronal differentiation 1
NF200: Neurofilament 200
NQO1: NAD(P)H quinone dehydrogenase 1
NP: Neural progenitors
NRF1: Nuclear respiratory factor 1
NFE2L2 (NRF2): Nuclear factor, erythroid 2-like 2
NSC: Neural stem cells generated from hiPSC
OXPHOS: Oxidative phosphorylation
PHB: Prohibitin
POLG: DNA polymerase gamma, catalytic subunit
POU5F1: POU class 5 homeobox 1
PPARs: Peroxisome proliferator-activated receptors
PPARA: Peroxisome proliferator-activated receptor alpha
PPARD: Peroxisome proliferator-activated receptor delta
PPARG: Peroxisome proliferator-activated receptor gamma
PPARGC1A: Peroxisome proliferator-activated receptor gamma coactivator 1 alpha (PGC-1α)
PPARGC1B: PPARG coactivator 1 beta
PPRE: Peroxisome proliferator response element
PQQ: Pyrroloquinoline quinone
PRKAA1: Protein kinase AMP-activated catalytic subunit alpha 1
RABEP2: Rabaptin, RAB GTPase-binding effector protein 2
ROS: Reactive oxygen species
RPLP0: Ribosomal protein lateral stalk subunit P0
RXRA: Retinoid X receptor alpha
RXRB: Retinoid X receptor beta
RXRG: Retinoid X receptor gamma
qRT-PCR: Quantitative reverse transcription PCR
S100B: S100 calcium-binding protein B
SCLO2B1: Solute carrier organic anion transporter family, member 2B1
SDHA: Succinate dehydrogenase [ubiquinone] flavoprotein subunit, mitochondrial
SERPINA1: Serpin family A number 1
SIRT1: Sirtuin 1
SLC1A1: Solute carrier family 17 member 7
SLC1A2: Solute carrier family 1 member 2
SLC1A3: Solute carrier family 1 member 3
SLC6A4: Solute carrier family 6 member 4
SLC17A7: Solute carrier family 17 member 7
SOX2: Transcription factor SOX-2
TBP: TATA box-binding protein
TCA: Tricarboxylic acid cycle
TFAM: Transcription factor A mitochondrial precursor
TH: Tyrosine hydroxylase
THRB: Thyroid hormone receptor beta
TUBB3: Beta tubulin class III (BTUB)
UBC: Ubiquitin C
ZNF324B: Zinc finger protein 324B
