# Supplementary material for: Bezafibrate Upregulates Mitochondrial Biogenesis and Influence Neural Differentiation of Human-Induced Pluripotent Stem Cells
Source: Mol Neurobiol. 2018 Oct 13;56(6):4346–63. doi: 10.1007/s12035-018-1368-2 (PMC6505510; doi:10.1007/s12035-018-1368-2)
Supplement: Supplementary file 2 — (PDF 429 kb) [file 12035_2018_1368_MOESM2_ESM.pdf]

GeneMANIA report

Created on : 3 August 2018 14:07:22
Last database update : 13 March 2017 00:00:00
Application version : 3.6.0

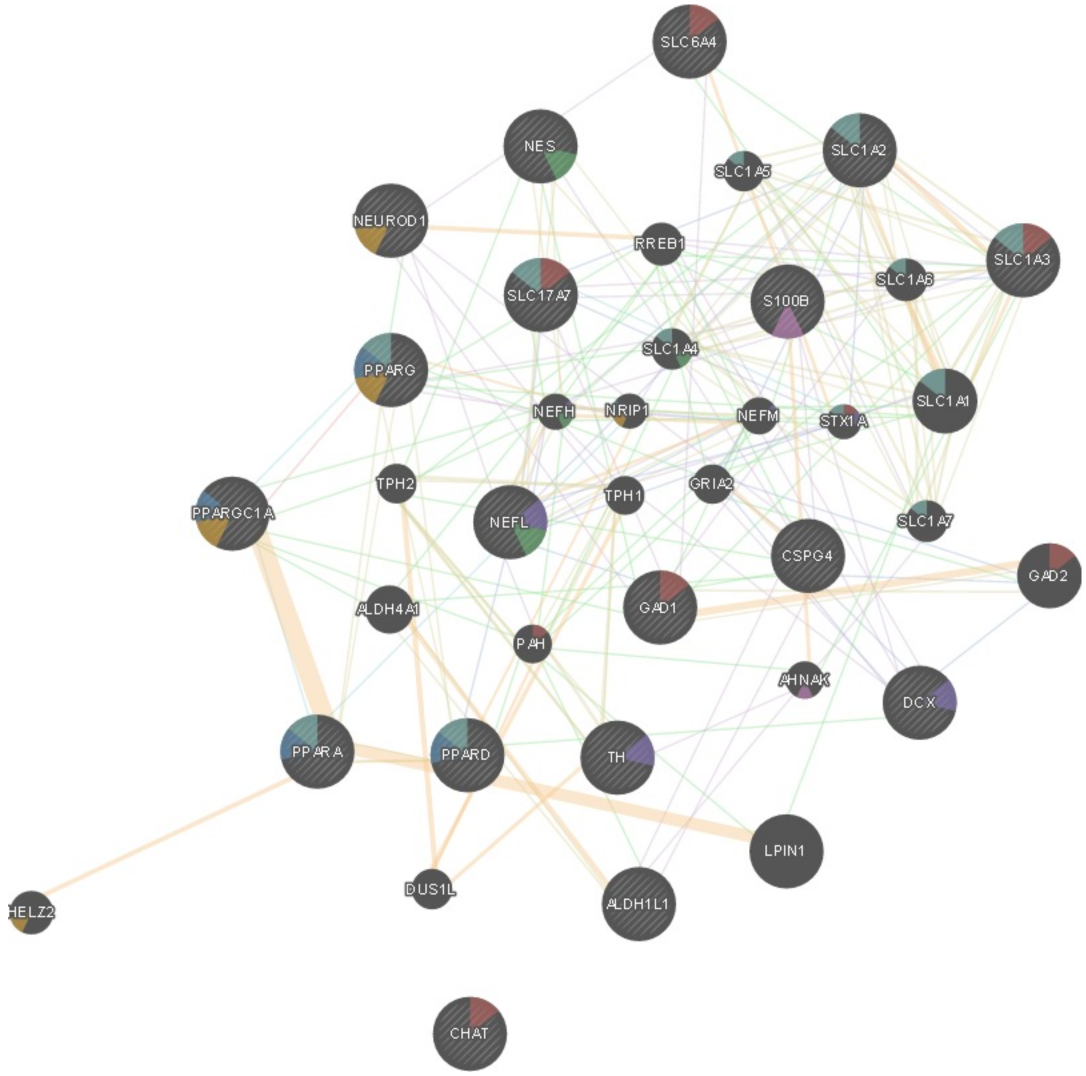

Networks

- Predicted
- Co-expression
- Shared protein domains
- Co-localization
- Physical Interactions
- Pathway
- Genetic Interactions

Functions

- regulation of neurotransmitter levels
- neuron projection
- intermediate filament cytoskeleton
- S100 protein binding
- transcription coactivator activity
- intracellular receptor signaling pathway
- carboxylic acid transport

Search parameters

Organism Homo sapiens (human)

Genes CHAT , DCX , SLC1A3 , SLC17A7 , SLC6A4 , PPARGC1A , SLC1A2 , PPARD , GAD1 , PPARG , NES , ALDH1L1 , S100B , NEUROD1 , CSPG4 , TH , PPARA , NEFL

Network weighting Automatically selected weighting method

Networks

A

Abu-Odeh-Aqeilan-2014 , Agrawal-Sedivy-2010 , Aichem-Groettrup-2012 , Albers-Koegl-2005 , Alexandru-Deshaies-2008 , Alizadeh-Staudt-2000 , Andresen-Flores-Morales-2014 , Arbuckle-Grant-2010 , Arroyo-Aloy-2014 , Arroyo-Aloy-2015

B

Bahr-Bowler-2013 , Bailey-Hieter-2015 , Bandyopadhyay-Ideker-2010 , Bantscheff-Drewes-2011 , Barr-Knapp-2009 , Barrios-Rodiles-Wrana-2005 , Behrends-Harper-2010 , Behzadnia-Lührmann-2007 , Bennett-Harper-2010 , Benzinger-Hermeking-2005 , Berggård-James-2006 , Bett-Hay-2013 , Bhatnagar-Attie-2014 , Bild-Nevins-2006 B , BIOGRID-SMALL-SCALE-STUDIES , BIOGRID-SMALL-SCALE-STUDIES , Blandin-Richard-2013 , Blomen-Brummelkamp-2015 , Blomen-Brummelkamp-2015 , Bogachek-Weigel-2014 , Boldrick-Relman-2002 , Bonacci-Soubeyran-2014 , Bouwmeester-Superti-Furga-2004 , Brajenovic-Drewes-2004 , Brehme-Superti-Furga-2009 , Bruderer-Hay-2011 , Burington-Shaughnessy-2008 , Butland-Hayden-2014 , Byron-Humphries-2012

C

Cai-Conaway-2007 , Camargo-Brandon-2007 , Campos-Reinberg-2015 , Cao-Chinnaiyan-2014 , Carmon-Liu-2014 , CELL_MAP , Chen-Brown-2002 , Chen-Ge-2013 , Chen-Huang-2014 , Chen-Zhang-2013 , Christianson-Kopito-2011 , Cloutier-Coulombe-2013 , Colland-Gauthier-2004 , Corominas-Iakoucheva-2014 , Couzens-Gingras-2013 , Cox-Rizzino-2013 , Coyaud-Raught-2015

D

Danielsen-Nielsen-2011 , Dart-Wells-2015 , de Hoog-Mann-2004 , Diner-Cristea-2015 , Dobbin-Giordano-2005 , Drissi-Boisvert-2015 , Dyer-Sobral-2010

E

Emanuele-Elledge-2011 , Emdal-Olsen-2015 , Ewing-Figeys-2007

F

Fenner-Prehn-2010 , Floyd-Pagliarini-2016 , Foerster-Ritter-2013 , Fogeron-Lange-2013 , Foster-Marshall-2013 , Freibaum-Taylor-2010

G

G

Gabriel-Baumgrass-2016 , Galligan-Howley-2015 , Gao-Reinberg-2012 , Gautier-Hall-2009 , Giannone-Liu-2010 , Glatter-Gstaiger-2009 , Gloeckner-Ueffing-2007 , Goehler-Wanker-2004 , Golebiowski-Hay-2009 , Goudreault-Gingras-2009 , Grant-2010 , Greco-Cristea-2011 , Grossmann-Stelzl-2015 , Guarani-Harper-2014 , Gupta-Pelletier-2015

H

Hanson-Clayton-2014 , Hauri-Gstaiger-2013 , Havrylov-Redowicz-2009 , Havugimana-Emili-2012 , Hayes-Urbé-2012 , Hegele-Stelzl-2012 A , Hegele-Stelzl-2012 B , Hein-Mann-2015 , Hill-Livingston-2014 , HUMANCYC , Humphries-Humphries-2009 , Hutchins-Peters-2010 , Huttlin-Gygi-2015

I

I2D-BIND-Fly2Human , I2D-BIND-Mouse2Human , I2D-BIND-Rat2Human , I2D-BIND-Worm2Human , I2D-BIND-Yeast2Human , I2D-BioGRID-Fly2Human , I2D-BioGRID-Mouse2Human , I2D-BioGRID-Rat2Human , I2D-BioGRID-Worm2Human , I2D-BioGRID-Yeast2Human , I2D-Chen-Pawson-2009-PiwiScreen-Mouse2Human , I2D-Formstecher-Daviet-2005-Embryo-Fly2Human , I2D-Giot-Rothbert-2003-Low-Fly2Human , I2D-INNATEDB-Mouse2Human , I2D-IntAct-Fly2Human , I2D-IntAct-Mouse2Human , I2D-IntAct-Rat2Human , I2D-IntAct-Worm2Human , I2D-IntAct-Yeast2Human , I2D-Krogan-Greenblatt-2006-Core-Yeast2Human , I2D-Krogan-Greenblatt-2006-NonCore-Yeast2Human , I2D-Li-Vidal-2004-CORE-1-Worm2Human , I2D-Li-Vidal-2004-non-core-Worm2Human , I2D-Manual-Mouse2Human , I2D-Manual-Rat2Human , I2D-MGI-Mouse2Human , I2D-MINT-Fly2Human , I2D-MINT-Mouse2Human , I2D-MINT-Rat2Human , I2D-MINT-Worm2Human , I2D-MINT-Yeast2Human , I2D-Ptacek-Snyder-2005-Yeast2Human , I2D-Tarassov-PCA-Yeast2Human , I2D-Tewari-Vidal-2004-TGFb-Worm2Human , I2D-vonMering-Bork-2002-High-Yeast2Human , I2D-vonMering-Bork-2002-Low-Yeast2Human , I2D-vonMering-Bork-2002-Medium-Yeast2Human , I2D-Wang-Orkin-2006-EScmplx-Mouse2Human , I2D-Wang-Orkin-2006-EScmplxlow-Mouse2Human , I2D-Yu-Vidal-2008-GoldStd-Yeast2Human , IMID , Ingham-Pawson-2005 , Innocenti-Brown-2011 , INTERPRO , IREF-BIND , IREF-BIOGRID , IREF-DIP , IREF-HPRD , IREF-INTACT , IREF-MATRIXDB , IREF-MPPI , IREF-PUBMED , IREF-SMALL-SCALE-STUDIES , IREF-SMALL-SCALE-STUDIES

J

Jeronimo-Coulombe-2007 , Jin-Pawson-2004 , Johnson-Kerner-Wichterle-2015 , Johnson-Shoemaker-2003 , Jones-MacBeath-2006 , Joshi-Cristea-2013 , Jäger-Krogan-2011

K

Kahle-Zoghbi-2011 , Kaltenbach-Hughes-2007 , Katsogiannou-Rocchi-2014 , Kim-Gygi-2011 , Kim-Major-2015 , Kneissl-Grummt-2003 , Koch-Hermeking-2007 ,

K

Kotlyar-Jurisica-2015 , Kristensen-Foster-2012 , Kärblane-Sarmiento-2015 , Kırlı-Görlich-2015

L

Lambert-Gingras-2015 , Lamoliatte-Thibault-2014 , Lau-Ronai-2012 , Lee-Songyang-2011 , Lehner-Sanderson-2004 A , Lehner-Sanderson-2004 B , Leng-Wang-2014 , Leung-Jones-2014 , Li-Chen-2015 , Li-Dorf-2011 A , Li-Dorf-2011 B , Li-Dorf-2014 , Li-Haura-2013 , Lim-Zoghbi-2006 , Lin-Smith-2010 , Lipp-Guthrie-2015 , Liu-Wang-2012 , Llères-Lamond-2010 , Loch-Strickler-2012 , Low-Heck-2014 , Lu-Zhang-2013 , Luo-Elledge-2009

M

Mak-Moffat-2010 , Mallon-McKay-2013 , Malovannaya-Qin-2010 , Markson-Sanderson-2009 , Maréchal-Zou-2014 , Matsumoto-Nakayama-2005 , McCracken-Blencowe-2005 , McFarland-Nussbaum-2008 , Meek-Piwnica-Worms-2004 , Milev-Mouland-2012 , Miyamoto-Sato-Yanagawa-2010 , Murakawa-Landthaler-2015

N

Nakayama-Ohara-2002 , Nakayasu-Adkins-2013 , Napolitano-Meroni-2011 , Narayan-Bennett-2012 , Nathan-Goldberg-2013 , NCI_NATURE , Neganova-Lako-2011 , Newman-Keating-2003 , Nicholson-Hupp-2014 , Noble-Diehl-2008

O

Oliviero-Cagney-2015 , Olma-Pintard-2009 , Oláh-Ovádi-2011 , Oshikawa-Nakayama-2012 , Ouyang-Gill-2009

P

Panigrahi-Pati-2012 , Papp-Lamia-2015 , Perez-Hernandez-Yáñez-Mó-2013 , Perou-Botstein-1999 , Perou-Botstein-2000 , Persaud-Rotin-2009 , Petschnigg-Stagljar-2014 , PFAM , Phillips-Corn-2013 , Pichlmair-Superti-Furga-2011 , Pichlmair-Superti-Furga-2012 , Pilot-Storck-Goillot-2010 , Povlsen-Choudhary-2012

R

Ramachandran-LaBaer-2004 , Raman-Harper-2015 , Ramaswamy-Golub-2001 , Ravasi-Hayashizaki-2010 , REACTOME , Reinke-Keating-2013 , Reyniers-Taymans-2014 , Richter-Chrzanowska-Lightowlers-2010 , Rieger-Chu-2004 , Rolland-Vidal-2014 , Rosenwald-Staudt-2001 , Roth-Zlotnik-2006 , Roux-Burke-2012 , Rowbotham-Mermoud-2011 , Roy-Pardo-2014 , Roy-Parent-2013 , Rual-Vidal-2005 A , Rual-Vidal-2005 B

S

Sang-Jackson-2011 , Sato-Conaway-2004 , Schadt-Shoemaker-2004 , Scholz-Taylor-2016 , Singh-Moore-2012 , Smirnov-Cheung-2009 , So-Colwill-2015 , Soler-López-Aloy-2011 , Sowa-Harper-2009 , Stehling-Lill-2012 , Stehling-Lill-2013 , Stelzl-Wanker-2005 , Stes-Gevaert-2014 , Stuart-Kim-2003 , Suter-Wanker-2013

T

Taipale-Lindquist-2012 , Taipale-Lindquist-2014 , Takahashi-Conaway-2011 , Tarallo-Weisz-2011 , Tatham-Hay-2011 , Teixeira-Gomes-2010 , Thalappilly-Dusetti-2008 , Thompson-Luchansky-2014 , Tong-Moran-2014 , Toyoshima-Grandori-2012 , Tsai-Cristea-2012

U

Udeshi-Carr-2012

V

---

van Wijk-Timmers-2009 , Vandamme-Angrand-2011 , Varjosalo-Gstaiger-2013 , Varjosalo-Superti-Furga-2013 , Venkatesan-Vidal-2009 , Vermeulen-Mann-2010 , Vinayagam-Wanker-2011 , Virok-Fülöp-2011 , Vizeacoumar-Moffat-2013

W

Wagner-Choudhary-2011 , Wallach-Kramer-2013 , Wan-Emili-2015 , Wang-Balch-2006 , Wang-Cheung-2015 , Wang-He-2008 , Wang-Maris-2006 , Wang-Xu-2015 , Wang-Yang-2011 , Weimann-Stelzl-2013 A , Weimann-Stelzl-2013 B , Weinmann-Meister-2009 , Wen-Wu-2014 , Whisenant-Salomon-2015 , Wilker-Yaffe-2007 , Willingham-Muchowski-2003 , Witt-Labeit-2008 , Wong-O'Bryan-2012 , Woods-Monteiro-2012 , Woodsmith-Sanderson-2012 , Wu-Garvey-2007 , Wu-Li-2007 , Wu-Ma-2012 , Wu-Stein-2010 , Wu-Stein-2010

X

Xiao-Lefkowitz-2007 , Xie-Cong-2013 , Xie-Green-2012 , Xu-Ye-2012

Y

Yang-Chen-2010 , Yatim-Benkirane-2012 , Yu-Chow-2013 , Yu-Vidal-2011

Z

Zanon-Pichler-2013 , Zhang-Shang-2006 , Zhang-Zou-2011 , Zhao-Krug-2005 , Zhao-Yang-2011 , Zhou-Conrads-2004 , Zhou-Hanemann-2016

Genes

Gene	Description	Rank
CHAT	choline O-acetyltransferase [Source:HGNC Symbol;Acc:HGNC:1912]	N/A
NES	nestin [Source:HGNC Symbol;Acc:HGNC:7756]	N/A
SLC1A2	solute carrier family 1 member 2 [Source:HGNC Symbol;Acc:HGNC: 10940]	N/A
SLC17A7	solute carrier family 17 member 7 [Source:HGNC Symbol;Acc:HGNC: 16704]	N/A
SLC1A3	solute carrier family 1 member 3 [Source:HGNC Symbol;Acc:HGNC: 10941]	N/A
SLC6A4	solute carrier family 6 member 4 [Source:HGNC Symbol;Acc:HGNC: 11050]	N/A
TH	tyrosine hydroxylase [Source:HGNC Symbol;Acc:HGNC:11782]	N/A
CSPG4	chondroitin sulfate proteoglycan 4 [Source:HGNC Symbol;Acc:HGNC: 2466]	N/A
ALDH1L1	aldehyde dehydrogenase 1 family member L1 [Source:HGNC Symbol; Acc:HGNC:3978]	N/A
DCX	doublecortin [Source:HGNC Symbol;Acc:HGNC:2714]	N/A
PPARD	peroxisome proliferator activated receptor delta [Source:HGNC Symbol; Acc:HGNC:9235]	N/A
NEFL	neurofilament, light polypeptide [Source:HGNC Symbol;Acc:HGNC: 7739]	N/A
GAD1	glutamate decarboxylase 1 [Source:HGNC Symbol;Acc:HGNC:4092]	N/A
PPARG	peroxisome proliferator activated receptor gamma [Source:HGNC Symbol;Acc:HGNC:9236]	N/A
PPARGC1A	PPARG coactivator 1 alpha [Source:HGNC Symbol;Acc:HGNC:9237]	N/A
NEUROD1	neuronal differentiation 1 [Source:HGNC Symbol;Acc:HGNC:7762]	N/A
S100B	S100 calcium binding protein B [Source:HGNC Symbol;Acc:HGNC: 10500]	N/A
PPARA	peroxisome proliferator activated receptor alpha [Source:HGNC Symbol; Acc:HGNC:9232]	N/A
LPIN1	lipin 1 [Source:HGNC Symbol;Acc:HGNC:13345]	1
GAD2	glutamate decarboxylase 2 [Source:HGNC Symbol;Acc:HGNC:4093]	2
SLC1A1	solute carrier family 1 member 1 [Source:HGNC Symbol;Acc:HGNC: 10939]	3

Gene	Description	Rank
ALDH4A1	aldehyde dehydrogenase 4 family member A1 [Source:HGNC Symbol; Acc:HGNC:406]	4
HELZ2	helicase with zinc finger 2 [Source:HGNC Symbol;Acc:HGNC:30021]	5
RREB1	ras responsive element binding protein 1 [Source:HGNC Symbol;Acc: HGNC:10449]	6
SLC1A6	solute carrier family 1 member 6 [Source:HGNC Symbol;Acc:HGNC: 10944]	7
SLC1A7	solute carrier family 1 member 7 [Source:HGNC Symbol;Acc:HGNC: 10945]	8
SLC1A4	solute carrier family 1 member 4 [Source:HGNC Symbol;Acc:HGNC: 10942]	9
DUS1L	dihydrouridine synthase 1 like [Source:HGNC Symbol;Acc:HGNC:30086]	10
SLC1A5	solute carrier family 1 member 5 [Source:HGNC Symbol;Acc:HGNC: 10943]	11
TPH2	tryptophan hydroxylase 2 [Source:HGNC Symbol;Acc:HGNC:20692]	12
GRIA2	glutamate ionotropic receptor AMPA type subunit 2 [Source:HGNC Symbol;Acc:HGNC:4572]	13
TPH1	tryptophan hydroxylase 1 [Source:HGNC Symbol;Acc:HGNC:12008]	14
PAH	phenylalanine hydroxylase [Source:HGNC Symbol;Acc:HGNC:8582]	15
AHNAK	AHNAK nucleoprotein [Source:HGNC Symbol;Acc:HGNC:347]	16
NEFM	neurofilament, medium polypeptide [Source:HGNC Symbol;Acc:HGNC: 7734]	17
NEFH	neurofilament, heavy polypeptide [Source:HGNC Symbol;Acc:HGNC: 7737]	18
NRIP1	nuclear receptor interacting protein 1 [Source:HGNC Symbol;Acc: HGNC:8001]	19
STX1A	syntaxin 1A [Source:HGNC Symbol;Acc:HGNC:11433]	20

Networks

**Predicted**	63.88%

I2D-INNATEDB-Mouse2Human — 51.40%

InnateDB: facilitating systems-level analyses of the mammalian innate immune response. Lynn et al (2008). *Mol Syst Biol*

Predicted with 1,451 interactions from I2D

Wu-Stein-2010 — 12.48%

A human functional protein interaction network and its application to cancer data analysis. Wu et al (2010). *Genome Biol*

Predicted with 87,829 interactions from supplementary material

**Co-expression**	17.75%

Wang-Maris-2006 — 5.02%

Integrative genomics identifies distinct molecular classes of neuroblastoma and shows that multiple genes are targeted by regional alterations in DNA copy number. Wang et al (2006). *Cancer Res*

Co-expression with 264,023 interactions from GEO

Chen-Brown-2002 — 3.16%

Gene expression patterns in human liver cancers. Chen et al (2002). *Mol Biol Cell*

Co-expression with 282,241 interactions from supplementary material

Noble-Diehl-2008 — 2.00%

Regional variation in gene expression in the healthy colon is dysregulated in ulcerative colitis. Noble et al (2008). *Gut*

Co-expression with 661,539 interactions from GEO

Ramaswamy-Golub-2001 — 1.82%

Multiclass cancer diagnosis using tumor gene expression signatures. Ramaswamy et al (2001). *Proc Natl Acad Sci U S A*

Co-expression with 275,113 interactions from supplementary material

Bild-Nevins-2006 B — 1.67%

Oncogenic pathway signatures in human cancers as a guide to targeted therapies. Bild et al (2006). *Nature*

Co-expression with 280,683 interactions from GEO

Wu-Garvey-2007 — 1.65%

The effect of insulin on expression of genes and biochemical pathways in human skeletal muscle. Wu et al (2007). *Endocrine*

Co-expression with 267,109 interactions from GEO

Perou-Botstein-2000 — 1.52%

Molecular portraits of human breast tumours. Perou et al (2000). *Nature*

Co-expression with 185,068 interactions from supplementary material

Dobbin-Giordano-2005 — 0.92%

Interlaboratory comparability study of cancer gene expression analysis using oligonucleotide microarrays. Dobbin et al (2005). *Clin Cancer Res*

Co-expression with 444,931 interactions from GEO

**Shared protein domains**	12.06%

INTERPRO — 11.60%

Shared protein domains with 608,863 interactions from InterPro

Shared protein domains 12.06%

PFAM 0.45%

Shared protein domains with 457,054 interactions from Pfam

Co-localization 3.71%

Schadt-Shoemaker-2004 2.30%

A comprehensive transcript index of the human genome generated using microarrays and computational approaches. Schadt et al (2004). *Genome Biol*

Co-localization with 60,126 interactions from GEO

Johnson-Shoemaker-2003 1.41%

Genome-wide survey of human alternative pre-mRNA splicing with exon junction microarrays. Johnson et al (2003). *Science*

Co-localization with 426,332 interactions from GEO

Physical Interactions 1.41%

IREF-INTACT 1.41%

Physical Interactions with 56,297 interactions from iRefIndex

Pathway 0.86%

Wu-Stein-2010 0.86%

A human functional protein interaction network and its application to cancer data analysis. Wu et al (2010). *Genome Biol*

Pathway with 78,010 interactions from supplementary material

Genetic Interactions 0.33%

Lin-Smith-2010 0.33%

A genome-wide map of human genetic interactions inferred from radiation hybrid genotypes. Lin et al (2010). *Genome Res*

Genetic Interactions with 4,820,370 interactions from supplementary material
